# Research trends on neutrophil extracellular traps in ischemic stroke: a scientific metrology study

**DOI:** 10.3389/fphar.2025.1537566

**Published:** 2025-04-11

**Authors:** Yaji Xu, Xingyu Gong, Yilin Wang, Xinyu Liu, Haomou Pu, Hongjie Jiang, Xiaoping Yu

**Affiliations:** ^1^ School of Preclinical Medicine, Chengdu University, Chengdu, China; ^2^ Development and Regeneration Key Laboratory of Sichuan Province, Chengdu Medical College, Chengdu, China; ^3^ School of Public Health, Chengdu University of Traditional Chinese Medicine, Chengdu, China

**Keywords:** neutrophil extracellular traps, ischemic stroke, bibliometric analysis, CiteSpace, VOSviewer, topic modelling

## Abstract

**Background:**

Ischemic stroke (IS) remains a leading global cause of mortality and chronic disability. Neutrophil extracellular traps (NETs), recently identified as neutrophil-derived structures that trap and neutralize pathogens, have increasingly drawn attention for their involvement in IS pathogenesis. Despite a surge in related research, no bibliometric analyses have yet examined the knowledge framework and trends within this emerging field. Here, we conducted a systematic bibliometric analysis to map the research landscape and identify current and potential hotspots regarding NETs in IS.

**Methods:**

Relevant literature published between 2014 and 2024 was systematically retrieved from the Web of Science Core Collection (WoSCC) database. Multiple analytical tools, including CiteSpace, VOSviewer, R package “bibliometrix,” and Python scripts, were employed to explore publication trends, academic collaborations, prominent research themes, and emerging topics.

**Results:**

Ninety-two publications were eligible for bibliometric assessment, supplemented by an additional latent Dirichlet allocation (LDA)-based topic analysis of 4,504 documents. China (30 publications) and the United States (23 publications) emerged as leading countries in terms of research output, with global collaboration networks predominantly centering around the United States. Noteworthy contributions also arose from European institutions, particularly Katholieke Universiteit Leuven and Karolinska Institutet. The leading authors identified were Desilles, Jean-Philippe, Ho-Tin-Noé, Benoit, and Mazighi, Mikael. Journals such as *Stroke*, *Annals of Neurology*, and *Nature Communications* significantly influenced this domain. Three main research hotspots emerged: (1) “promoting effect of protein arginine deiminase 4 (PAD4) in NET formation,” (2) “cell-free DNA as a biomarker for disease diagnosis,” and (3) “influence of platelets and thrombosis on NETs-related diseases.”

**Conclusion:**

Our study offers an extensive overview of existing literature and evolving research trajectories concerning NETs in IS, providing researchers with clear insights into current trends and future investigative directions. Nevertheless, our study has limitations—including dependence on a single database (WoSCC), restriction to English-language publications, and inherent constraints of the LDA methodology—that merit consideration in interpreting these findings.

## 1 Introduction

Ischemic stroke (IS), characterized by high morbidity, mortality, and severe long-term disabilities (Faustino et al., 2019; Hurford et al., 2020), remains a significant global health challenge, accounting for approximately 86.8% of all stroke cases (Koton et al., 2022; Tu et al., 2023). Pathologically, IS arises from the abrupt interruption of cerebral blood flow, typically due to thrombotic or embolic events, leading to acute neuronal injury and substantial neurological deficits (Tuo et al., 2022; Qin et al., 2022). Such neurological impairments markedly reduce patients’ quality of life. Current acute ischemic stroke (AIS) treatment relies on thrombolytic therapy and mechanical thrombectomy. These treatments have strict time limits: intravenous thrombolysis must be given within 4.5 h, and mechanical thrombectomy must be performed within 24 h. As a result, fewer than 10% of patients can benefit from these interventions (Wu et al., 2020; Hilkens et al., 2024). Consequently, there is an urgent need to identify novel therapeutic strategies.

Despite considerable advances in our understanding of IS, the underlying mechanisms remain incompletely elucidated. Pathophysiological processes involve complex interactions among oxidative stress, neuroinflammation, apoptosis, and necrosis, collectively exacerbating neuronal damage (Roth et al., 2018; Li et al., 2022b; Han et al., 2023; Gao et al., 2024). Recent research has indicated that neutrophil extracellular traps (NETs) may play a pivotal role in IS pathology (Kang et al., 2020; Denorme et al., 2022; Dhanesha et al., 2022), warranting comprehensive investigation as potential therapeutic targets.

Neutrophils are a fundamental component of the innate immune system, serving as primary defenders against infectious pathogens (Liew and Kubes, 2019). Brinkmann et al. first described NETs in 2004 as web-like structures composed of decondensed DNA intertwined with various granular proteins, released by activated neutrophils into the extracellular environment (Brinkmann et al., 2004; Fousert et al., 2020). NET formation primarily occurs via the NADPH oxidase-dependent reactive oxygen species (ROS) signaling pathway, highlighting neutrophils’ critical roles in infection management and inflammation regulation (Amulic et al., 2012).

In the context of IS, NETs exhibit dual and paradoxical roles (Manda-Handzlik and Demkow, 2019). Neutrophils can polarize into two distinct phenotypes: N1, a pro-inflammatory subtype that exacerbates neuronal injury, and N2, which exhibits protective properties (Hou et al., 2019; Cai et al., 2020). Under ischemic conditions, imbalances favoring the N1 phenotype increase NET formation, which may aggravate brain injury by intensifying thrombotic and inflammatory responses at lesion sites (Ducroux et al., 2018; Lim et al., 2020; Li et al., 2022a). Elevated NETs levels have been documented in the blood of AIS patients, suggesting their potential as early diagnostic markers, indicators of disease severity, and guides for therapeutic interventions (Vallés et al., 2017; Genchi et al., 2021). Nonetheless, current findings remain inconsistent, and a systematic bibliometric evaluation of the research landscape related to NETs in IS remains lacking.

Bibliometrics is a scientific metrology study employing statistical and computational methods to elucidate the structure, characteristics, and development of literature within specific research fields (Ellegaard and Wallin, 2015). By rapidly and precisely identifying critical research hotspots and future trajectories, bibliometrics has become an invaluable tool across numerous disciplines, including IS research (Wang et al., 2023a; Chen et al., 2023a; Zhu et al., 2023; Jiang et al., 2024). In this study, we employed bibliometric analysis to delineate research trends and highlight potential directions for investigations into NETs in IS over the past decade. Our goal is to clarify the evolution, current status, and future directions of this rapidly advancing field.

## 2 Methods

### 2.1 Data connection and search strategy

**FIGURE 1:**
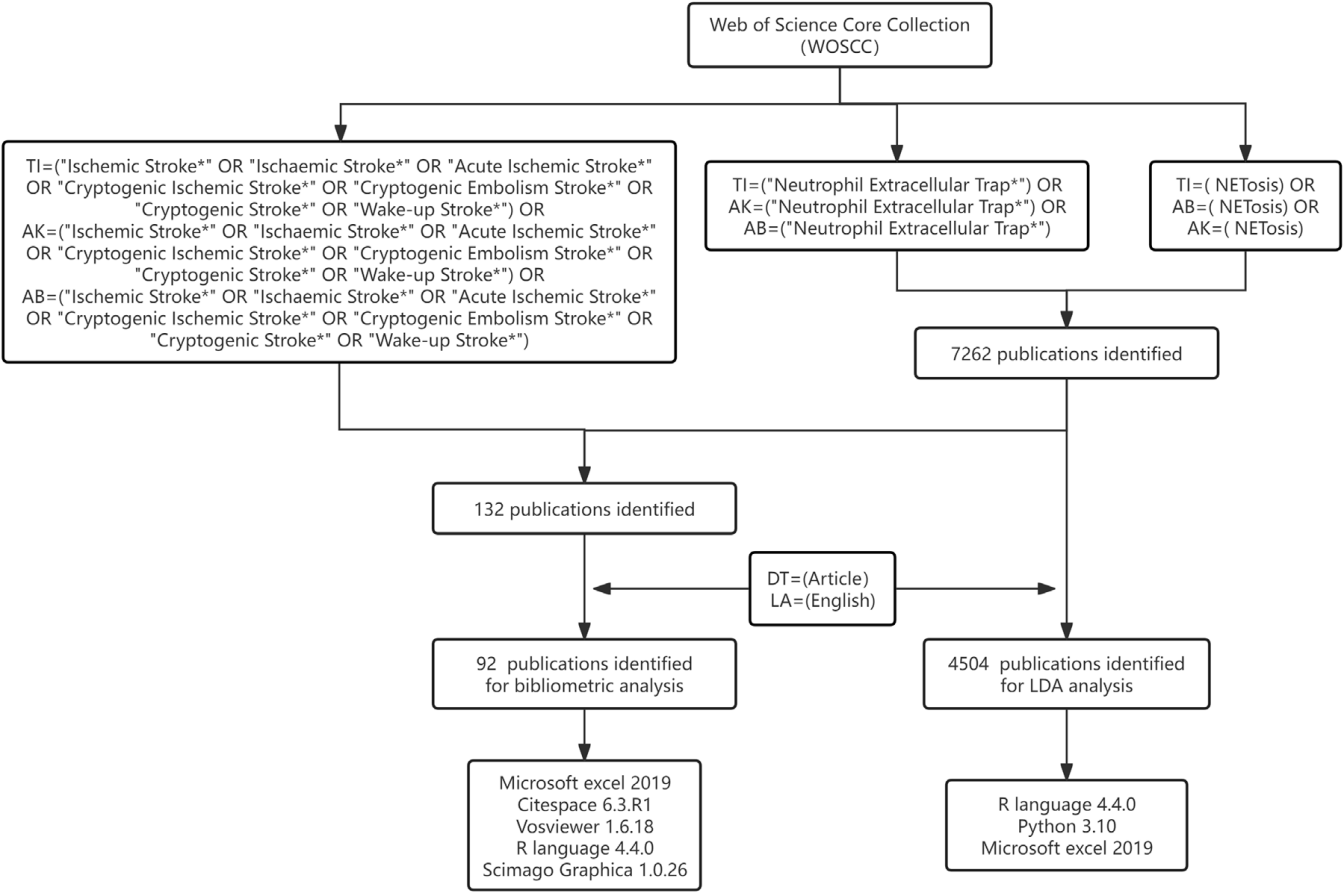
Flow chart displaying the search strategy and analytical process for IS-NETs research.

The Web of Science Core Collection (WoSCC) is a premier bibliometric database providing high-quality, structured data from over 13,000 reputable scholarly journals, making it highly suitable for bibliometric analyses (Singh et al., 2021; Yang et al., 2022). We conducted a comprehensive literature search in the WoSCC database on 12 August 2024, utilizing the following search terms: TI or AB or AK= (“Ischemic Stroke” OR “Ischaemic Stroke” OR “Acute lschemic Stroke” OR “Cryptogenic lschemic Stroke” OR “Cryptogenic Embolism Stroke” OR “Cryptogenic Stroke” OR “Wake-up Stroke”) and (“Neutrophil Extracellular Trap” or “NETosis”); TI or AB or AK = “Neutrophil Extracellular Trap” or “NETosis.” The search criteria were restricted to “Articles” published in English. The retrieved publications were exported in multiple formats, including plain text, Excel, BibTeX, and tab-delimited files. A detailed search strategy is provided in Supplementary Table 1. Ultimately, 92 and 4,504 records were chosen for further text analysis. The methodological framework employed in this study is illustrated in Figure 1.

### 2.2 Analysis and visualization

The software tools used for the literature dosage analysis were VOSviewer (version 1.6.20), CiteSpace (version 6.2.R2), Rstudio (version 4.4.0), and Python (version 3.10). VOSviewer facilitated the creation and visualization of co-authorship, co-occurrence, and co-citation networks acroos references, journals, and authors (van Eck and Waltman, 2010). CiteSpace, a specialized citation visualization software, was employed to forecast research trends by clustering keywords and visualizing journal dual-map overlays and keyword networks (Wu et al., 2021).

Additionally, latent Dirichlet allocation (LDA), a robust topic modeling approach widely applied across disciplines such as marketing, economics, and bioinformatics (Chen et al., 2022a), was utilized to identify prominent themes within large textual datasets. First, data preprocessing involved removing stop words. The optimal number of topics was determined by evaluating paired cosine distance (cao_juan_2009), Kullback-Leibler divergence (arun_2010), and the model coherence metric (coherence_mimno_2011) (Supplementary Figure 1). Following this evaluation, we selected 30 key topics based on consensus among the three methods. Topic labels were generated through independent labeling and classification methods, which focus on the top twenty weighted subject words and the top ten relevant articles. Two annotators independently label and classify the data. Cross-validation was subsequently conducted, and discrepancies were resolved through consultation with a third expert. Ultimately, this process led to the final set of topics and genes associated with NETs in IS. The 4,504 publications relating to NETs and NETosis were then imported into Python (version 3.10) for detailed LDA and gene analyses.

## 3 Results

### 3.1 Annual trends in publication

**FIGURE 2:**
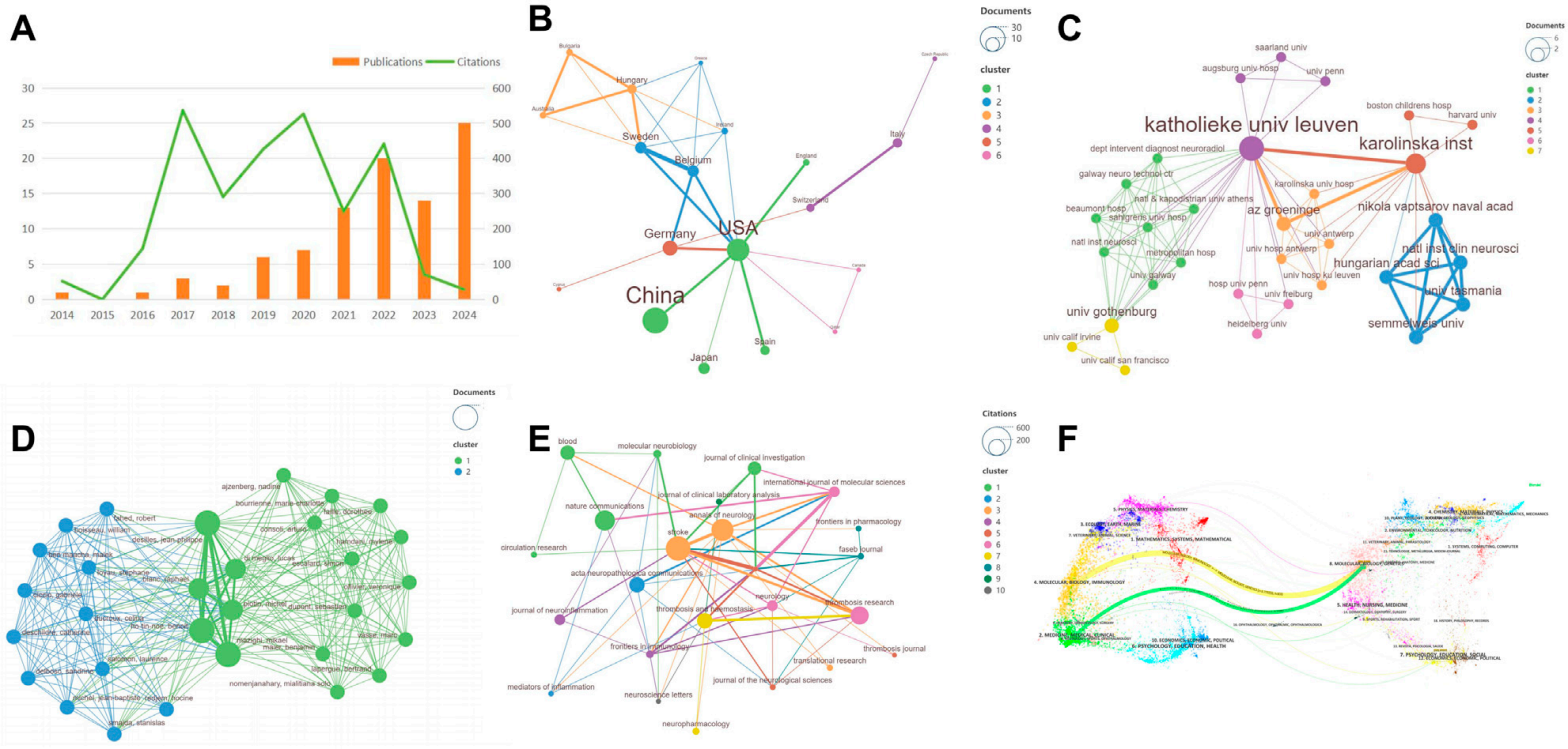
Annual publication trends and co-authorship network cluster analysis. **(A)** Publications and citations of IS-NETs over time; **(B)** Visualization of collaborative research efforts among countries; **(C)** Visualizations of co-authorship between institutions; **(D)** Visualization of author collaborations; **(E)** Visualization of journal co-citation networks with over 15 citations, with distinct clusters represented by different colors; **(F)** The dual-map overlap of journals.

Research on ischemic stroke (IS), neutrophil extracellular traps (NETs), and NETosis has undergone significant development over the past decade. This bibliometric analysis systematically evaluated publication trends from 2014 to 2024 (Figure 2A). Results reveal a consistent increase in research output and scholarly attention. Initially modest from 2014 to 2018, publication volume steadily rose thereafter, peaking in 2021 and again in 2024, indicating renewed scientific interest. However, citation frequency fluctuated until 2022, followed by a rapid decline, suggesting a potential delay in recognizing the full impact of recent publications.

### 3.2 Analysis of countries and institutions

China and the United States have led global research on NETs in IS, with China producing 30 publications and the United States 23 publications. Both countries dominate in total publication count and overall citations; However, their normalized citation impact remains relatively modest, suggesting quantity does not necessarily equate to influence. Notably, several European countries, such as Sweden and Spain, have demonstrated significant research impact despite fewer publications (Figure 2B; Table 1). The global collaboration network predominantly centers on the United States, particularly through strong partnerships with China and Germany.

**TABLE 1:** Top 10 most cited countries, institutions, authors, and journals.

Rank		Citations	Publications	Average citations
Country				
1	China	642	30	21.40
2	United States	566	23	24.61
3	Germany	206	10	20.60
4	Belgium	357	6	59.50
5	Japan	47	6	7.83
6	Sweden	498	6	83.00
7	Hungary	64	4	16.00
8	Italy	59	4	14.75
9	Spain	269	4	67.25
10	Switzerland	54	3	18.00
Institutions				
1	Katholieke Univ Leuven	357	6	59.50
2	Karolinska Inst	486	4	121.50
3	Az Groeninge	349	2	174.50
4	Hungarian Acad Sci	54	2	27.00
5	Natl Inst Clin Neurosci	54	2	27.00
6	Nikola Vaptsarov Naval Acad	54	2	27.00
7	Semmelweis Univ	54	2	27.00
8	Univ Gothenburg	12	2	6.00
9	Univ Tasmania	54	2	27.00
10	Augsburg Univ Hosp	3	1	3.00
Authors				
1	Desilles, Jean-Philippe	251	3	83.67
2	Ho-Tin-Noé, Benoit	251	3	83.67
3	Mazighi, Mikael	251	3	83.67
4	Blanc, Raphael	246	2	123.00
5	Di Meglio, Lucas	246	2	123.00
6	Piotin, Michel	246	2	123.00
7	Ben Maacha, Malek	234	1	234.00
8	Boisseau, William	234	1	234.00
9	Ciccio, Gabriele	234	1	234.00
10	Delbosc, Sandrine	234	1	234.00
Journals				
1	*Stroke*	413	6	68.83
2	*Annals Of Neurology*	333	1	333.00
3	*Nature Communications*	279	1	279.00
4	*Thrombosis Research*	226	6	37.67
5	*Acta Neuropathologica Communications*	164	2	82.00
6	*Thrombosis And Haemostasis*	163	2	81.50
7	*Blood*	146	2	73.00
8	*Journal Of Clinical Investigation*	127	1	127.00
9	*Neurology*	80	1	80.00
10	*International Journal Of Molecular Sciences*	79	5	15.80

European institutions significantly influence this research landscape. Katholieke Univ Leuven (six publications) and Karolinska Inst (four publications) are the leading institutions by publication output. However, Az Groeninge in Belgium exhibits the highest average citation rate, indicating its high research quality and influence. Additionally, our findings suggest that collaborative networks among these European institutions frequently align geographically (Figure 2C).

### 3.3 Analysis of authors and journals

A total of 716 authors have contributed significantly to NET-related IS research. The top three most productive authors—Jean-Philippe Desilles, Benoit Ho-Tin-Noé, and Mikael Mazighi—each contributed three publications with a cumulative citation count of 251. Although Malek Ben Maacha et al. achieved higher average citation rate of 234, the total number of publications by individual authors remains relatively limited (Table 1). Co-authorship analysis (Figure 2D) identifies a core collaborative network centered around Jean-Philippe Desilles and colleagues.

Fifty-nine distinct journals have published articles on NETs in IS, highlighting the interdisciplinary nature of the field. Journals such as *Stroke*, *Annals of Neurology*, and *Nature Communications* hold considerable influence, as indicated by citation analysis (Table 1). Co-citation network analysis positions *Stroke* as the most interconnected journal. This suggests that it may serve as a key bridge across various research areas (Figure 2E).

Figure 2F presents a dual-map overlay of journals generated by Citespace, depicting the primary citation pathways between citing and cited journals. Journals within medicine/clinical/healthcare and molecular/biology/immunology cite literature from molecular/biology/genetics journals. This illustrates the interdisciplinary cross-fertilization characterizing contemporary medical research and underscores foundational research’s broad impact.

### 3.4 Analysis of references and keywords

**FIGURE 3:**
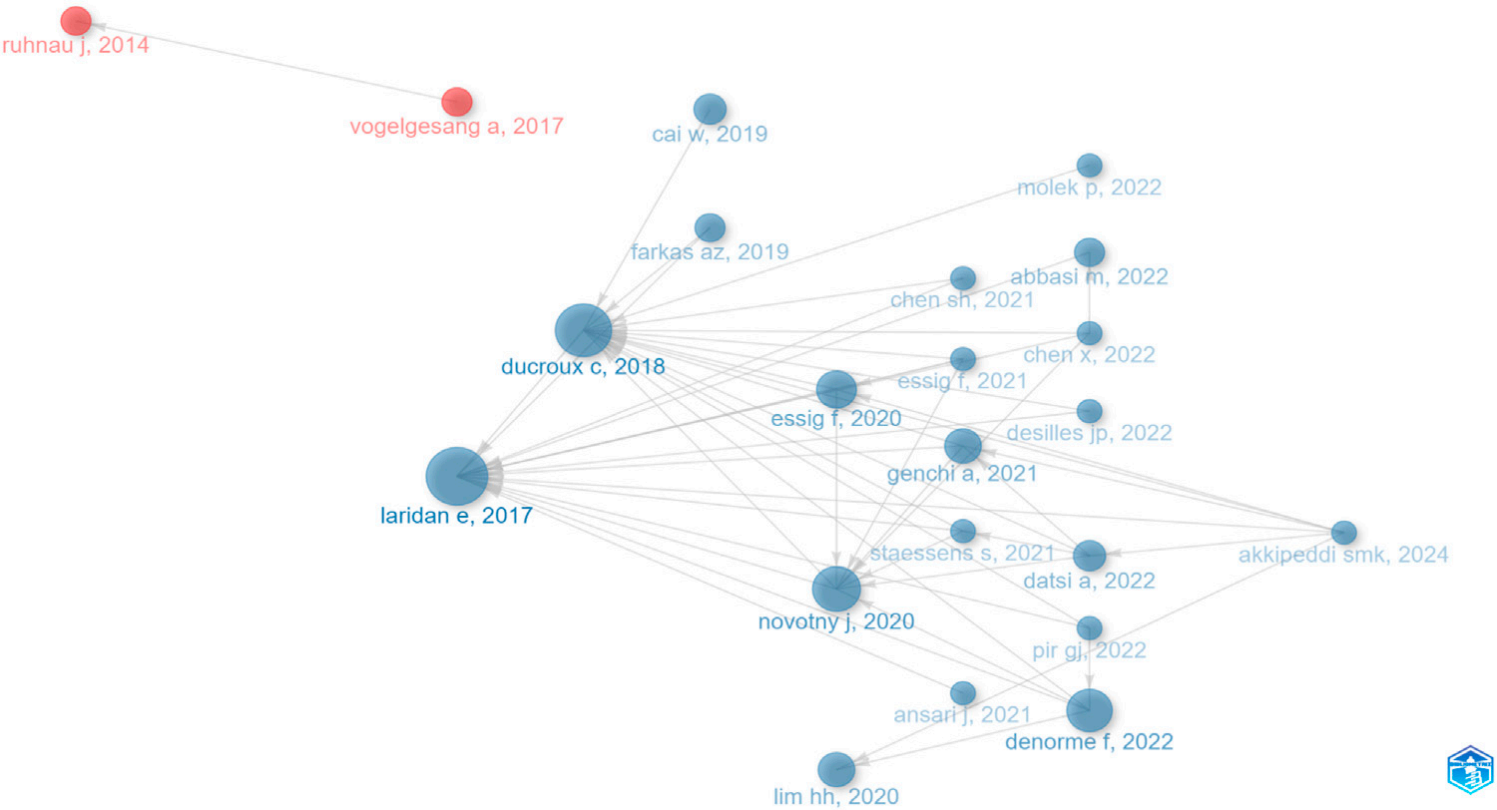
Historiographic map illustrating the evolution of reference documents.

To better understand the evolution of research on NETs in IS, a historiographic timeline was constructed using R software to identify major shifts in focus and key milestones (Figure 3; Supplementary Table 2). The progression clearly outlines initial explorations into pathological mechanisms transitioning toward therapeutic applications.

Between 2014 and 2017, research focused on neutrophil and monocyte function post-IS. Subsequent studies (2017–2019) examined NETs’ roles in stroke-related thrombosis. From 2019 to 2022, the scope broadened to include NETs as diagnostic biomarkers, their involvement across various clot types, and associations with clinical outcomes. Recent studies (2023–2024) have further specialized, investigating COVID-19 impacts on clot composition and exploring connections between NETs and collateral circulation, advancing the understanding of IS pathology and potential treatments.

Table 2 summarizes the top 10 most-cited publications. It reveals a shift from foundational mechanistic insights toward clinical relevance. Initially (2016–2017), studies validated NET presence and fundamental roles in IS. Later publications increasingly targeted clinical implications, assessing NETs in prognosis (2017) and therapeutic responsiveness (2018). Furthermore, research expanded from thrombotic involvement (2017) to broader implications in vascular remodeling (2020), cerebral injury (2019, 2022), and related areas. Collectively, these influential studies emphasize NETs’ increasing importance in IS, moving progressively from basic biology to practical clinical strategies.

**TABLE 2:** The top 10 most cited publications relevant to IS-NETs research.

Rank	Title	Citation	Year	Journal	JCR	IF (2023)
1	Neutrophil extracellular traps in ischemic stroke thrombi	333	2017	Ann Neurol	Q1	8.1
2	Neutrophil extracellular traps released by neutrophils impair revascularization and vascular remodeling after stroke	279	2020	Nat Commun	Q1	14.7
3	Thrombus Neutrophil Extracellular Traps Content Impair tPA-Induced Thrombolysis in Acute Ischemic Stroke	234	2018	Stroke	Q1	7.8
4	Neutrophil extracellular traps are increased in patients with acute ischemic stroke: prognostic significance	154	2017	Thromb Haemost	Q2	5
5	Neutrophil extracellular trap induced by HMGB1 exacerbates damages in the ischemic brain	134	2019	Acta Neuropathol Commun	Q2	6.2
6	NETosis promotes cancer-associated arterial microthrombosis presenting as ischemic stroke with troponin elevation	130	2016	Thromb Res	Q3	3.7
7	Neutrophil extracellular traps regulate ischemic stroke brain injury	127	2022	J Clin Invest	Q1	13.3
8	Neutrophil extracellular traps promote tPA-induced brain hemorrhage via cGAS in mice with stroke	90	2021	Blood	Q1	21
9	Pharmacological Modulation of Neutrophil Extracellular Traps Reverses Thrombotic Stroke tPA (Tissue-Type Plasminogen Activator) Resistance	84	2019	Stroke	Q1	7.8
10	Thrombus NET content is associated with clinical outcome in stroke and myocardial infarction	80	2020	Neurology	Q1	7.7

**FIGURE 4:**
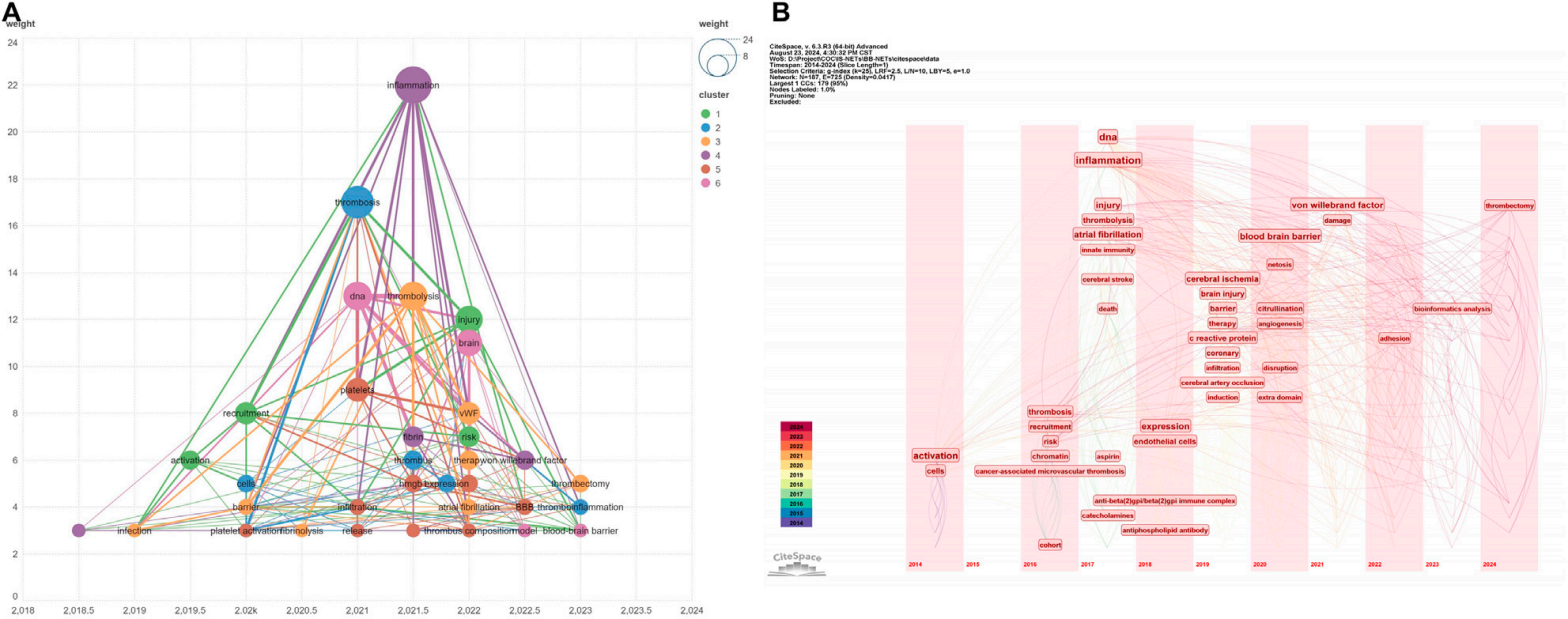
Keyword analysis visualizations in IS-NETs research. **(A)** Temporal overlay visualization of keyword co-occurrence; **(B)** Keyword timeline visualization.

Keyword analysis via VOSviewer (Figure 4A) and CiteSpace (Figure 4B) revealed research focal points. Recently emphasized keywords include “thrombectomy,” “thromboinflammation,” and “blood-brain barrier.” Additionally, emerging research themes such as “bioinformatics analysis,” “von Willebrand factor,” and “thrombectomy” reflect the field’s evolving and increasingly specialized landscape.

### 3.5 Topic modeling

**FIGURE 5:**
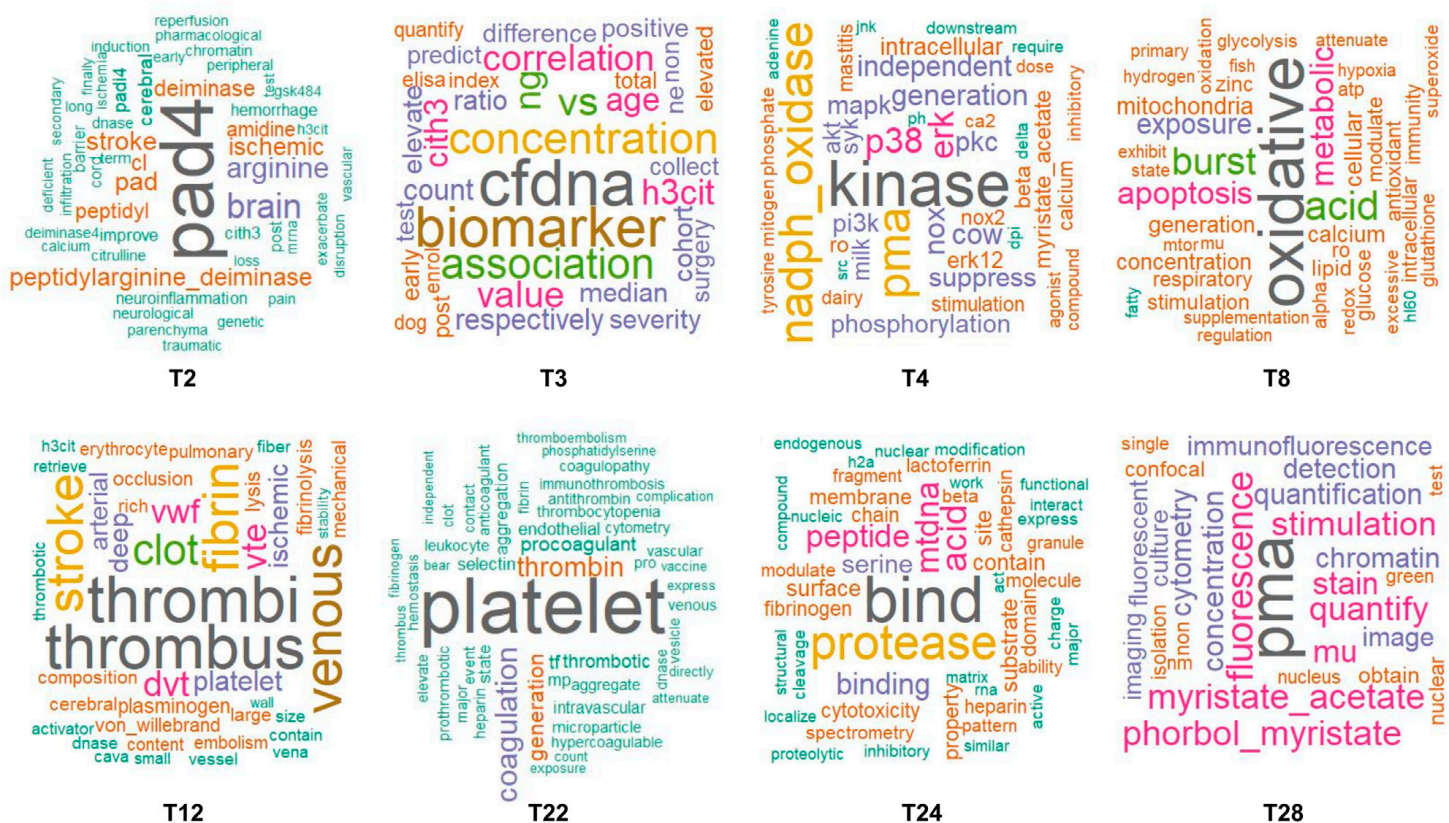
World cloud of NETs Research in Medical Biosciences and IS.

Due to the limited volume of existing research specifically addressing NETs in IS, we employed LDA topic modeling to analyze 4,504 publications related broadly to NETs and NETosis to explore new research directions. Following established analytical criteria, 30 key topics were identified (Supplementary Figure 2), which were subsequently named based on their primary terms and related articles (Supplementary Tables 3, 4). These topics were categorized into two broad themes: the role of NETs in disease, and biomedical research on NETs related specifically to IS (Table 3). We visualized eight relevant biomedical topics associated with NETs in IS through word cloud analyses (Figure 5). These central themes included “pad4,” “cfdna,” “kinase,” “oxidative,” “thrombi,” “platelet,” “bind,” and “pma.” For example, topic 2 prominently featured “pad4,” reflecting its central role in NET formation in IS, accompanied by secondary terms such as “neuroinflammation”, “barrier”, and “hemorrhage.” These terms collectively highlight research on NETs’ influence on neuroinflammation, blood-brain barrier integrity, hemorrhagic complications, and potential pharmacological interventions targeting NET formation. Topic 3 emphasized cell-free DNA (cfDNA) as a potential biomarker for disease diagnosis and prognosis, with frequent terms including “biomarker,” “concentration”, and “association,” underscoring the diagnostic potential of cfDNA. Terms such as “nucleosome,” “H3Cit,” and “CitH3” indicated a specific focus on DNA markers linked to NETs.

**TABLE 3:** Results of topic modeling analysis for NETs and NETosis research.

The role of NETs in diseases
T1 The Impact of NETs on Diabetic Wound Healing and Inflammation
T5 The Role of NETs in Intestinal Inflammation and Barrier Dysfunction
T6 The Role of NETs in Cancer Progression and Metastasis
T7 The Role of Nanoparticles and NETs in Systemic Inflammatory Diseases
T9 The Function of Low-Density Neutrophils in Immunosenescence and Disease
T10 The Therapeutic Role of NETs in LPS-Induced Acute Lung Injury
T11 The Role of TLR and NETs in Skin Inflammation and Stem Cell-Based Therapies for Inflammatory Diseases
T13 The Defensive Mechanisms of NETs in Fungal and Bacterial Infections
T14 The Mechanistic Study of NETs in Antimicrobial Resistance
T15 NETs Gene Signatures and Prognostic Biomarkers in Cancer
T16 The Future of Clinical Translation of NETs
T17 The Impact of Environmental Exposure and Therapeutic Interventions on NETs in Respiratory Diseases
T18 The Interaction between NETs Degradation and Streptococcal Nucleases
T19 The Impact of NETs in COVID-19
T20 NETs and RA
T21 NETs and Host-Parasite Interactions
T23 The Role of NETs in Liver Injury and Ischemia/Reperfusion Injury
T25 Endothelial Dysfunction, Antiphospholipid Syndrome, and the Formation of NETs
T26 The Role of NETs in CAD and AMI
T27 The Role of NETs in Systemic Lupus Erythematosus
T29 NETs and Sepsis
T30 The Role of Neutrophil Extracellular Traps in ANCA-Associated Vasculitis

Research on NETs in Medical Biosciences and IS
T2 The Effects of NETs on Cerebral Ischemia and Hemorrhage
T3 NETs Biomarkers and Prognosis
T4 The Signaling Pathways in NETs Formation
T8 The Role of Oxidative Stress and Mitochondrial Dynamics in NETs Formation
T12 The Mechanism and Composition Analysis of Thrombosis in IS
T22 The Role of NETs in Platelet Activation and Coagulation
T24 The Role of Polysialic Acid and Proteases in NETs Formation
T28 Advanced Techniques in the Quantification and Imaging of NETs

Note: RA: rheumatoid arthritis, CAD: coronary artery disease, AMI: acute myocardial infarction.

Topic 4 concentrated on signaling pathways underlying NET formation, evidenced by high-frequency terms such as “kinase,” “NADPH oxidase,” “ERK,” and “p38,” which reflect the involvement of kinases and oxidoreductases. This topic highlights research on signaling pathways, including MAPK, PI3K/AKT, and PKC, in regulating NET production. Topic 8 centered around oxidative stress and autophagy. Common terms like “oxidative,” “stress,” “autophagy,” and “mitochondrial” underscore the significance of oxidative stress and mitochondrial function in generating NETs. Topic 12 addresses thrombosis and the role of fibrin networks in NETs-associated diseases. Dominant terms like “thrombi,” “thrombus,” “venous,” and “fibrin” emphasize thrombus formation, particularly in venous thrombosis and fibrin networks. The terms “stroke” and “ischemic” indicate that the studies also investigate the role of NETs in IS.

### 3.6 Gene analysis

We analyzed and extracted relevant genetic elements from the eight identified themes, resulting in 312 associated genes (Supplementary Table 5). By comparing genes occurring more than twice within our dataset to those documented in 92 IS-NETs-related publications, we identified 26 overlapping genes (Table 4). These shared genes highlight several research topics, such as “NETs in medical and biological sciences,” which remain underexplored within IS-specific NETs literature, despite their potential significance. This finding highlights critical opportunities for innovation and points toward future research directions. Furthermore, our LDA topic modeling identified several areas corresponding with current research hotspots, reinforcing their significance in IS research.

**TABLE 4:** Common genes identified in NETs, NETosis, and IS-NETs pathways.

Gene names	First appearance year in Part 1	Freq in Part 1	Topic	Freq in Part 2
TLR4	2007	15	T4	8
TF	2012	5	T12	1
PAD4	2013	30	T2	12
vWF	2014	32	T12	10
ERK	2015	9	T4	2
PKC	2016	2	T4	1
NLRP3	2017	2	T12	1
H2B	2017	3	T24	1
IL-18	2017	3	T3	1
NOX	2018	3	T4	1
HMGB1	2018	12	T12	5
CXCL7	2019	3	T22	1
MPO	2019	18	T3	9
CXCL4	2019	5	T22	1
STING	2019	9	T2	3
TNF-Alpha	2019	5	T4	3
S100A8	2021	5	T3	1
S100A9	2021	6	T3	1
CXCR4	2021	2	T2	2
IL-6	2021	3	T3	1
CCL2	2022	2	T2	1
MAPK	2022	5	T4	1
JAK2	2022	3	T22	1
CCL5	2023	2	T2	1
IL-8	2023	2	T3	1
CITH3/H3CIT	2017	65	T3	15

Note: Freq: frequency, Part 1 = Article for Bibliometric analysis inNETs, and NETosis, Part 2 = Article for Topic Cluster “Research on NETs, in Medical Biosciences and IS.”

The identified genes are pivotal in multiple biological pathways and mechanisms associated with NET formation and function, including inflammation, coagulation, oxidative stress, and cell signaling. Key genes like CITH3/H3CIT, PAD4, and MPO are directly involved in NET formation, making them essential to NETs research. Coagulation-related genes such as von Willebrand factor (vWF) and Tissue Factor are crucial for NETs-associated thrombosis. Mediators like TLR4 and HMGB1 initiate NET formation and subsequent inflammatory responses. Inflammatory regulators, including S100A8/A9, TNF-α, IL-6, and IL-8, modulate inflammation related to NET activity. Signaling pathway-associated genes like ERK, STING, MAPK, and JAK2 potentially govern NET formation via various cell signaling cascades. Additionally, chemokines CXCL4 and CXCL7 participate in immune cell recruitment processes connected to NETs, while NLRP3 is associated with NET-induced inflammasome activation.

## 4 Discussion

This study presents a comprehensive bibliometric analysis of research on neutrophil extracellular traps (NETs) in ischemic stroke (IS), covering publications from 1 January 2014, to 12 August 2024. This is, to our knowledge, the first systematic bibliometric evaluation of NETs-related research within the context of IS. We employed qualitative, quantitative, and integrative approaches to identify current research trends, hotspots, and potential future directions in this field.

### 4.1 General information

Our bibliometric analysis reveals a steady annual growth in research publications on NETs in IS, despite the relatively limited total publication volume. Although there was a slight decrease in 2023, a surge is projected for 2024, highlighting the substantial research potential and increasing interest in this area. However, recent declines in citation frequency (Figure 2A) post 2022 suggest that factors such as publication lag, research funding fluctuations, and shifting scientific priorities may be influencing the perceived impact of this body of work. Additionally, improving research quality remains needed to enhancing the scholarly impact of future publications.

China and the United States dominate this research domain, likely due to their extensive research initiatives and strong financial support systems. Nevertheless, normalized citation metrics for China are comparatively lower, possibly reflecting weaker international collaboration networks, limited cross-institutional referencing, or publication in journals with moderate impact; only two of the ten most cited publications since 2020 have originated from Chinese researchers. European institutions, particularly Katholieke Universiteit Leuven and Karolinska Institutet, have substantial publication outputs driven by geographical proximity facilitating collaboration (Figure 2C). Conversely, limited cooperation between Asian and European institutions presents a barrier to international research collaboration and academic advancement. To overcome these challenges, enhancing international research collaboration through strategic funding initiatives and institutional partnerships is essential.

Journal analysis indicates that *Stroke*, *Annals of Neurology*, and *Nature Communications* lead in contributions in this field, all ranked within the Q1 category according to Journal Citation Reports (JCR) (Table 2). Among the top ten cited journals, three journals possess an impact factor (IF) over 10, and nine have IF values greater than 5, highlighting the high quality of research published. Prominent neuroscience journals, including *Stroke*, *Annals of Neurology*, and *Acta Neuropathologica Communications*, have significantly advanced understanding of NETs in IS, reinforcing NETs’ importance as biomarkers and potential therapeutic targets (Laridan et al., 2017; Ducroux et al., 2018; Kang et al., 2020).

The most cited publication, authored by Laridan et al. from KU Leuven Campus Kulak Kortrijk, titled “Neutrophil Extracellular Traps in Ischemic Stroke Thrombi” appeared in *Annals of Neurology* 7 years ago (Laridan et al., 2017). This foundational study demonstrated H3Cit, a NET-specific marker, in nearly all analyzed thrombi from IS patients, correlating NETs with cardiac etiology and specific histopathological features. These insights have significantly advanced our understanding of IS pathophysiology and therapeutic opportunities targeting NETs. The second most influential study by Kang et al., published in Nature Communications in 2020, elucidated how NETs impede vascular remodeling following IS (Kang et al., 2020). This study showed that the increased expression of the enzyme PAD4 exacerbated blood-brain barrier disruption and impaired neurovascular repair, ultimately worsening patient outcomes. The third most referenced article, published 6 years ago in *Stroke* by Celina Ducroux et al. from the Université Paris Diderot, is titled “Thrombus Neutrophil Extracellular Traps Content Impair tPA-Induced Thrombolysis in Acute Ischemic Stroke” (Ducroux et al., 2018). It suggested that NETs within thrombi may contribute to thrombolytic resistance in IS, hindering successful reperfusion despite mechanical thrombectomy or pharmacological thrombolysis treatments. Importantly, this study highlighted recombinant DNase1 as a promising therapeutic adjunct to accelerate tissue plasminogen activator (t-PA)-mediated thrombolysis. Collectively, these three articles emphasized the therapeutic potential of targeting NETs in IS and highlight the importance of further research exploring NET-driven pathological mechanisms to enhance clinical outcomes.

### 4.2 The hotspots and the frontiers

Keywords and word cloud analyses could offer valuable insights into emerging trends within NET-related ischemic stroke (IS) research, though the literature remains relatively sparse. To address this gap, we applied latent Dirichlet allocation (LDA) topic modeling to a comprehensive dataset of 4,504 NET and NETosis-related publications. We identified eight distinct themes, represented through targeted word clouds, highlighting critical areas of current and potential future research (Figure 5). Topic 2 focuses on the essential role of PAD4 in NET formation and its implications for IS. As a key enzyme in the NETosis process, PAD4 expression and activity significantly increase following IS (Kim, et al., 2020), exacerbating blood-brain barrier disruption and hindering neurovascular regeneration, thus impairing post-stroke recovery (Kang et al., 2020). PAD4 overexpression also induces neuroinflammation and neuronal death via the STING-dependent IRE1α/ASK1/JNK signaling pathway, as demonstrated in traumatic brain injury models (Shi et al., 2023). PAD4 inhibition has yielded promising anti-inflammatory results in various conditions like asthma, spinal cord injury, and inflammatory bowel disease (IBD) (Feng et al., 2021; Chen et al., 2023c; Wang et al., 2024). Recent advancements in PAD4 inhibitors, especially in cancer research, present promising avenues for therapeutic applications in IS management (Deng et al., 2022).

Topic 3 emphasizes circulating cell-free DNA (cfDNA) as a diagnostic and prognostic biomarker. Elevated cfDNA originates from cellular apoptosis, necrosis, and secretion during pathological states, including inflammation, infection, and cancer (Chen et al., 2022b; Wang et al., 2022). It has been reported that 7-day cfDNA concentration is associated with clinical outcomes, particularly mortality rates in IS by cohort studies (Grosse et al., 2022). Additionally, cfDNA assists in evaluating short-term outcomes in AIS and the risk of hemorrhage post-thrombolysis (Orbán-Kálmándi et al., 2021b). NETs are associated with poor prognoses in patients with IS and may contribute to inflammation and thrombosis (Baumann et al., 2024). Therefore, integrating cfDNA with NET biomarkers may enhance our understanding of IS pathophysiology. Future applications of bioinformatics and machine learning may further optimize cfDNA and NETs biomarker usage for improved diagnostic accuracy and treatment monitoring (Wang et al., 2023b).

Topics 12 and 22 examine platelets’ roles and thrombotic mechanisms in NET-related diseases. Historically, thrombi predominantly comprise platelets, fibrin, and erythrocytes; however, increasing evidence highlights neutrophils’ contributions. Studies by Elodie et al. have documented NETs in thrombi and the presence of neutrophils in these clots, even if their numbers vary among IS patients (Laridan et al., 2017). Platelets are essential for clotting as they release cytokines and bioactive substances like CXCL4, which triggers NETosis—a critical process where neutrophils expel their DNA to ensnare pathogens and debris (Matsumoto et al., 2021; Jarrahi et al., 2023). In platelet-rich regions of IS clots, the composition becomes complex, including fibrin, von Willebrand factor (vWF), and DNA (Wang et al., 2018). Research shows von Willebrand factor (vWF) deficiency significantly reduces NET formation in IS models, identifying vWF as a potential therapeutic target (De Wilde et al., 2022).

Additionally, biomarkers reflective of NET levels, such as MPO, citH3, cfDNA, nucleosomes, and DNase-I, have predictive value in peripheral artery and coronary artery ischemic diseases (Bang et al., 2019; Orbán-Kálmándi et al., 2021a). In AIS patients, peripheral blood NETs and neutrophil counts significantly elevate within 24 h post-stroke, correlating with increased severity and poorer prognosis, further emphasizing NETs’ roles in ischemic pathophysiology and their potential for disease monitoring (Datsi et al., 2022; Huang et al., 2022; Liaptsi et al., 2023). Although data during the hyperacute phase of stroke are limited, animal models show dynamic NET changes, detectable at 6 h, peaking at 24 h, and decreasing at 48 h post-stroke (De Wilde et al., 2022). These observations demonstrate the utility of word cloud analyses for rapidly identifying emerging research trends on NETs in IS.

Importantly, several highly expressed genes significantly influence NET formation and function in IS. Elevated HMGB1 levels during the acute stroke phase correlate with worse outcomes by enhancing NET formation and recruitment, leading to neurofunctional deficits (Shan et al., 2022; Mu et al., 2024b). During acute inflammation, NET-microparticle complexes facilitated neutrophil recruitment through the HMGB1-TLR2/TLR4 signaling pathway (Wang et al., 2019). Furthermore, NETs intensify inflammatory responses via TLR4/HIF-1α signaling in choroidal endothelial cells (Zeng et al., 2023). The MPO-DNA, an accurate marker of NET formation, peaks around 24 h post-stroke, representing an optimal therapeutic window (Li et al., 2024).

Several important markers are closely tied to post-stroke inflammation. Notably, NODlike receptor family pyrin domain-containing 3 (NLRP3), the most extensively studied inflammasome component, can be activated by TLR ligands (Guo et al., 2015). There is emerging evidence that the activation of NLRP3 inflammasome is involved in NETosis. This activation may also contribute to the onset of thrombosis (Broz and Dixit, 2016). PAD4, vital for NETosis initiation, also participates in NLRP3 inflammasome activation, and NLRP3 gene ablation significantly decreases NET release (Münzer et al., 2021). NETs trigger caspase-1 activation within macrophages, further enhancing NLRP3 inflammasome activity (Kahlenberg et al., 2013). Research demonstrates that the NLRP3 inflammasome is essential for the release of NETs (Kumar et al., 2023), and consequently, dual-target inhibitors of NLRP3 and PAD4 offer promising clinical potential.

There is a complex relationship between NETs and oxidative stress, which leads to neuronal dysfunction and death through excessive ROS and reactive nitrogen species (RNS) production (Manoj et al., 2025). Excess ROS/RNS activation triggers mast cells and macrophages to release inflammatory cytokines such as TNF, IL-10, and IL-1 (Hotchkiss and Karl, 2003), which are critical for neutrophil adhesion and migration. Intracellular ROS mediates NETosis signaling, subsequently released NETs further amplify inflammatory cytokine production, creating a feedback loop that accelerates disease progression (Stoiber et al., 2015). Chen et al. suggested that regulating the Nrf2 signaling pathway can reduce mitochondrial oxidative damage, thereby providing neuroprotective effects (Chen et al., 2023b). Acknowledging the importance of interconnected mechanisms—like neutrophil extracellular traps, oxidative stress, and mitochondrial disfunction—can assist in identifying effective biomarkers and therapeutic targets for treating stroke.

Recent studies have shown that PAD4 inhibitor like BB-Cl-amidine (BBCA) could significantly reduce infarct volume and improve neurological function in MCAO rats. BBCA blocked the accumulation of citrullinated proteins (F95) during the acute phase and suppressed NETosis induction in the subacute phase (Seol et al., 2025). Similarly, the selective PAD4 inhibitor GSK484 reduced PAD4 activity by decreasing H3Cit+ EET production, suggesting its potential as an antithrombotic therapeutic agent (Martinod et al., 2024). To enhance drug delivery to ischemic regions, Mu, Q. et al. synthesized a neutrophil-targeting delivery system that responds to ROS and is loaded with GSK484. This innovative system could significantly boost the accumulation of GSK484 nanoparticles in brain lesions, reduce NET formation, and inhibit neuroinflammation (Mu et al., 2024a). Thus, PAD4 blockade forms a critical foundation for multi-mechanism targeted drug development. Still, clinical trials remain essential for validating these preclinical findings (Jiang et al., 2024). Despite the extensive research on IS, few clinical drug trials specifically target NETs. Notably, Fondation Ophtalmologique Adolphe de Rothschild initiated a phase 2 clinical trial (NCT04785066) evaluating the efficacy of NET-targeting Dornase alfa (Pulmozyme^®^) in IS, indicating significant progress toward clinical application.

### 4.3 Strengths and limitations

This study represents the first systematic bibliometric analysis to investigate research trends and developments concerning NETs in ischemic stroke (IS). By employing diverse bibliometric methods, our approach reduces analytical biases typically associated with conventional reviews. Furthermore, our findings reveal more distinct regional differences in research focus and output, highlighting the most influential authors, journals, and institutions. This information serves as a valuable reference for emerging researchers. Moreover, the LDA topic model identifies and visually represents eight core research topics related to NETs in biomedical and IS contexts, which helps future validation and exploration. Finally, the analysis of high-frequency genes, including PAD4, VWF, MPO, TLR4, and HMGB1, is closely related to NET formation, NLRP3 inflammasome activation, and oxidative stress pathways, thus identifying potential biomarkers and therapeutic targets.

Inevitably, this study has several limitations. Firstly, our analysis only included original English-language articles from the Web of Science Core Collection database, possibly limiting the comprehensiveness of our findings. Second, the relatively modest sample size of ninety-two publications may affect the robustness of our network analyses. Third, inherent methodological constraints of the LDA model—which segments sentences into individual words based solely on frequency—might not fully preserve the original context and nuanced significance of specific terms or phrases.

## 5 Conclusion

Our bibliometric analysis demonstrates a steadily growing interest in research exploring the interplay between NETs and IS. China and the United States are the leading nations significantly advancing research in this field. Three of the most cited papers discuss NETs and their contributions to thrombus formation, interference with thrombolytic therapies, and exacerbation of neurovascular injuries post-IS. Future research should focus on PAD4-mediated regulation of NET formation and the complex relationship between NETs and thrombosis, considering their dualistic nature in ischemic stroke pathology. Such research promises novel therapeutic strategies prioritizing neuroprotective outcomes. Targeting NETs thus emerges as a crucial area for future IS research, potentially improving patient prognosis and clinical management.

## Data Availability

The original contributions presented in the study are included in the article/Supplementary Material, further inquiries can be directed to the corresponding author.
