# Supplementary material for: Research trends on neutrophil extracellular traps in ischemic stroke: a scientific metrology study
Source: Front Pharmacol. 2025 Apr 11;16:1537566. doi: 10.3389/fphar.2025.1537566 (PMC12021921; doi:10.3389/fphar.2025.1537566)
Supplement: Supplementary file 1 [file DataSheet1.docx]

Supplementary Material

# Supplementary Tables

**Supplementary Table 1.** Search strategy

|  | **Search Query** | **Results** |
| --- | --- | --- |
| 1 | TI=("Ischemic Stroke*" OR "Ischaemic Stroke*" OR "Acute Ischemic Stroke*" OR "Cryptogenic Ischemic Stroke*" OR "Cryptogenic Embolism Stroke*" OR "Cryptogenic Stroke*" OR "Wake-up Stroke*") | 48512 |
| 2 | AB=("Ischemic Stroke*" OR "Ischaemic Stroke*" OR "Acute Ischemic Stroke*" OR "Cryptogenic Ischemic Stroke*" OR "Cryptogenic Embolism Stroke*" OR "Cryptogenic Stroke*" OR "Wake-up Stroke*") | 67433 |
| 3 | AK=("Ischemic Stroke*" OR "Ischaemic Stroke*" OR "Acute Ischemic Stroke*" OR "Cryptogenic Ischemic Stroke*" OR "Cryptogenic Embolism Stroke*" OR "Cryptogenic Stroke*" OR "Wake-up Stroke*") | 24584 |
| 4 | #1 OR #2 OR #3 | 93227 |
| 5 | TI=("Neutrophil Extracellular Trap*") | 3271 |
| 6 | AB=("Neutrophil Extracellular Trap*") | 5066 |
| 7 | AK=("Neutrophil Extracellular Trap*") | 2188 |
| 8 | #5 OR #6 OR #7 | 6499 |
| 9 | TI=("NETosis") | 612 |
| 10 | AB=("NETosis") | 1427 |
| 11 | AK=("NETosis") | 589 |
| 12 | #9 OR #10 OR #11 | 1932 |
| 13 | #8 OR #12 | 6499 |
| 14 | #4 AND #13 | 132 |
| 15 | LA=(English) | 78651640 |
| 16 | DT=(Article) | 51504715 |
| 17 | #14 AND #15 AND #16 | 92 |
| 18 | #13 AND #15 AND #16 | 4504 |
| **Note:** TI=title, AB=abstract, AK=author keyword, LA=language, DT=document type. | | |

**Supplementary Table 2.** Reference historiography

|  | **Title** | **DOI** | **Year** | **LCS** | **GCS** |
| --- | --- | --- | --- | --- | --- |
| 1 | Stroke Alters Respiratory Burst In Neutrophils And Monocytes | 10.1161/STROKEAHA.113.003342 | 2014 | 2 | 46 |
| 2 | Ischaemic Stroke And The Recanalization Drug Tissue Plasminogen Activator Interfere With Antibacterial Phagocyte Function | 10.1186/s12974-017-0914-6 | 2017 | 2 | 16 |
| 3 | Neutrophil Extracellular Traps In Ischemic Stroke Thrombi | 10.1002/ana.24993 | 2017 | 49 | 333 |
| 4 | Thrombus Neutrophil Extracellular Traps Content Impair Tpa-Induced Thrombolysis In Acute Ischemic Stroke | 10.1161/STROKEAHA.117.019896 | 2018 | 36 | 234 |
| 5 | Neutrophil Extracellular Traps In Thrombi Retrieved During Interventional Treatment Of Ischemic Arterial Diseases | 10.1016/j.thromres.2019.01.006 | 2019 | 2 | 47 |
| 6 | All Trans-Retinoic Acid Protects Against Acute Ischemic Stroke By Modulating Neutrophil Functions Through Stat1 Signaling | 10.1186/s12974-019-1557-6 | 2019 | 3 | 63 |
| 7 | Evaluation Of Neutrophil Extracellular Traps As The Circulating Marker For Patients With Acute Coronary Syndrome And Acute Ischemic Stroke | 10.1002/jcla.23190 | 2020 | 6 | 25 |
| 8 | Immunohistological Analysis Of Neutrophils And Neutrophil Extracellular Traps In Human Thrombemboli Causing Acute Ischemic Stroke | 10.3390/ijms21197387 | 2020 | 9 | 41 |
| 9 | Thrombus Net Content Is Associated With Clinical Outcome In Stroke And Myocardial Infarction | 10.1212/WNL.0000000000009532 | 2020 | 20 | 80 |
| 10 | Cerebral Thrombi Of Cardioembolic Etiology Have An Increased Content Of Neutrophil Extracellular Traps | 10.1016/j.jns.2021.117355 | 2021 | 6 | 24 |
| 11 | Detailed Histological Analysis Of A Thrombectomy-Resistant Ischemic Stroke Thrombus: A Case Report | 10.1186/s12959-021-00262-1 | 2021 | 1 | 16 |
| 12 | Netosis And Inflammasomes In Large Vessel Occlusion Thrombi | 10.3389/fphar.2020.607287 | 2021 | 1 | 23 |
| 13 | Neutrophils And Platelets: Immune Soldiers Fighting Together In Stroke Pathophysiology | 10.3390/biomedicines9121945 | 2021 | 1 | 5 |
| 14 | High Mobility Group Box 1 Protein In Cerebral Thromboemboli | 10.3390/ijms222011276 | 2021 | 1 | 3 |
| 15 | Stroke-Derived Neutrophils Demonstrate Higher Formation Potential And Impaired Resolution Of Cd66B+Driven Neutrophil Extracellular Traps | 10.1186/s12883-022-02707-0 | 2022 | 3 | 11 |
| 16 | Neutrophil Extracellular Traps Regulate Ischemic Stroke Brain Injury | 10.1172/JCI154225 | 2022 | 16 | 127 |
| 17 | Impact Of Covid-19 On Thrombus Composition And Response To Thrombolysis: Insights From A Monocentric Cohort Population Of Covid-19 Patients With Acute Ischemic Stroke | 10.1111/jth.15646 | 2022 | 1 | 12 |
| 18 | Platelet-Neutrophil Association In Nets-Rich Areas In The Retrieved Ais Patient Thrombi | 10.3390/ijms232214477 | 2022 | 1 | 8 |
| 19 | Leukocytes In Cerebral Thrombus Respond To Large-Vessel Occlusion In A Time-Dependent Manner And The Association Of Nets With Collateral Flow | 10.3389/fimmu.2022.834562 | 2022 | 1 | 7 |
| 20 | Diverse Thrombus Composition In Thrombectomy Stroke Patients With Longer Time To Recanalization | 10.1016/j.thromres.2021.11.018 | 2022 | 2 | 20 |
| 21 | Markers Of Net Formation And Stroke Risk In Patients With Atrial Fibrillation: Association With A Prothrombotic State | 10.1016/j.thromres.2022.02.025 | 2022 | 1 | 18 |
| 25 | Histone Content, And Thus Dna Content, Is Associated With Differential In Vitro Lysis Of Acute Ischemic Stroke Clots | 10.1016/j.jtha.2024.01.013 | 2024 | 1 | 2 |
| **LCS: Local Citation Count; GCS: Global Citation Count.** | | | | | |

**Supplementary Table 3.** The top 20 words most relevant to the topic

| **Topic** | **Word** | **Word Weight** |
| --- | --- | --- |
| **T1** | wound | 0.06057 |
|  | diabetic | 0.04551 |
|  | healing | 0.03976 |
|  | inflammasome | 0.03464 |
|  | gsdmd | 0.03355 |
|  | nlrp3 | 0.03247 |
|  | kidney | 0.03247 |
|  | renal | 0.02983 |
|  | pyroptosis | 0.02967 |
|  | diabetes | 0.02206 |
|  | dm | 0.0202 |
|  | aki | 0.01678 |
|  | diabete | 0.01477 |
|  | glucose | 0.01414 |
|  | il1_beta | 0.01119 |
|  | gasdermin | 0.01057 |
|  | delay | 0.009176 |
|  | caspase1 | 0.009021 |
|  | accelerate | 0.008555 |
|  | nod | 0.007468 |
| **T2** | pad4 | 0.1187 |
|  | brain | 0.04272 |
|  | arginine | 0.0314 |
|  | stroke | 0.02623 |
|  | pad | 0.02586 |
|  | ischemic | 0.02364 |
|  | peptidylarginine_deiminase | 0.02278 |
|  | deiminase | 0.02007 |
|  | cl | 0.01946 |
|  | peptidyl | 0.01798 |
|  | amidine | 0.01589 |
|  | cerebral | 0.0138 |
|  | padi4 | 0.01146 |
|  | cith3 | 0.0101 |
|  | improve | 0.00949 |
|  | hemorrhage | 0.00789 |
|  | barrier | 0.00752 |
|  | cord | 0.007151 |
|  | neuroinflammation | 0.007028 |
|  | post | 0.007028 |
| **T3** | cfdna | 0.02559 |
|  | biomarker | 0.0216 |
|  | concentration | 0.01602 |
|  | association | 0.0152 |
|  | vs | 0.01394 |
|  | nucleosome | 0.01296 |
|  | ng | 0.01291 |
|  | value | 0.01258 |
|  | correlation | 0.01236 |
|  | h3cit | 0.01209 |
|  | cith3 | 0.01061 |
|  | age | 0.01045 |
|  | respectively | 0.008861 |
|  | count | 0.008752 |
|  | ratio | 0.008642 |
|  | cohort | 0.00826 |
|  | median | 0.00815 |
|  | severity | 0.007986 |
|  | elevate | 0.007986 |
|  | positive | 0.007276 |
| **T4** | kinase | 0.04201 |
|  | nadph_oxidase | 0.02917 |
|  | pma | 0.02841 |
|  | erk | 0.01992 |
|  | p38 | 0.01992 |
|  | nox | 0.01535 |
|  | generation | 0.01525 |
|  | cow | 0.01525 |
|  | independent | 0.01459 |
|  | pkc | 0.01416 |
|  | suppress | 0.01307 |
|  | mapk | 0.01285 |
|  | pi3k | 0.0122 |
|  | phosphorylation | 0.01209 |
|  | syk | 0.01144 |
|  | milk | 0.01078 |
|  | akt | 0.01057 |
|  | intracellular | 0.01024 |
|  | phorbol_myristate | 0.01013 |
|  | myristate_acetate | 0.009913 |
| **T5** | intestinal | 0.04381 |
|  | colitis | 0.02584 |
|  | gut | 0.02455 |
|  | barrier | 0.02427 |
|  | ne | 0.01956 |
|  | western | 0.01799 |
|  | uc | 0.01756 |
|  | epithelial | 0.01685 |
|  | cd | 0.01599 |
|  | immunofluorescence | 0.01599 |
|  | ibd | 0.01457 |
|  | microbiota | 0.01385 |
|  | alleviate | 0.01342 |
|  | stain | 0.01214 |
|  | colon | 0.012 |
|  | mucosal | 0.01071 |
|  | blotting | 0.01043 |
|  | acid | 0.009287 |
|  | ulcerative | 0.008146 |
|  | infiltration | 0.008003 |
| **T6** | cancer | 0.133 |
|  | tumor | 0.1003 |
|  | metastasis | 0.05535 |
|  | breast | 0.02093 |
|  | progression | 0.01876 |
|  | microenvironment | 0.01793 |
|  | metastatic | 0.01726 |
|  | growth | 0.01568 |
|  | hcc | 0.01343 |
|  | gc | 0.01293 |
|  | survival | 0.01084 |
|  | carcinoma | 0.01051 |
|  | therapy | 0.01026 |
|  | invasion | 0.008677 |
|  | colorectal | 0.00851 |
|  | recurrence | 0.008427 |
|  | proliferation | 0.007176 |
|  | prognosis | 0.00701 |
|  | gastric | 0.00701 |
|  | liver | 0.006676 |
| **T7** | periodontitis | 0.02808 |
|  | crystal | 0.02459 |
|  | nanoparticle | 0.01799 |
|  | msu | 0.01696 |
|  | oral | 0.0167 |
|  | gout | 0.01592 |
|  | periodontal | 0.01424 |
|  | surface | 0.01333 |
|  | delivery | 0.01101 |
|  | particle | 0.01075 |
|  | therapy | 0.01049 |
|  | gingivali | 0.01049 |
|  | implant | 0.009584 |
|  | biomaterial | 0.009325 |
|  | resolution | 0.008291 |
|  | adjuvant | 0.007773 |
|  | property | 0.007644 |
|  | vaccine | 0.007644 |
|  | drug | 0.007515 |
|  | urate | 0.007127 |
| **T8** | oxidative | 0.0319 |
|  | stress | 0.0305 |
|  | autophagy | 0.0291 |
|  | mitochondrial | 0.02864 |
|  | acid | 0.01819 |
|  | burst | 0.01651 |
|  | metabolic | 0.01306 |
|  | apoptosis | 0.0126 |
|  | exposure | 0.0112 |
|  | mitochondria | 0.007842 |
|  | cellular | 0.007749 |
|  | generation | 0.007656 |
|  | calcium | 0.007563 |
|  | concentration | 0.007563 |
|  | lipid | 0.007003 |
|  | antioxidant | 0.006444 |
|  | ro | 0.00635 |
|  | respiratory | 0.006257 |
|  | modulate | 0.006257 |
|  | zinc | 0.006164 |
| **T9** | monocyte | 0.02711 |
|  | granulocyte | 0.02523 |
|  | csf | 0.01843 |
|  | density | 0.01739 |
|  | phenotype | 0.01625 |
|  | ldgs | 0.01446 |
|  | peripheral | 0.0138 |
|  | functional | 0.01323 |
|  | subset | 0.01314 |
|  | cytometry | 0.01115 |
|  | phagocytosis | 0.01078 |
|  | capacity | 0.00917 |
|  | differentiation | 0.00917 |
|  | exhibit | 0.007942 |
|  | number | 0.007848 |
|  | population | 0.007659 |
|  | aged | 0.007376 |
|  | chemotaxis | 0.007281 |
|  | age | 0.007092 |
|  | distinct | 0.006998 |
| **T10** | lps | 0.04245 |
|  | ali | 0.03255 |
|  | lp | 0.02876 |
|  | ard | 0.02044 |
|  | attenuate | 0.01791 |
|  | suppress | 0.01665 |
|  | pulmonary | 0.01623 |
|  | infiltration | 0.01391 |
|  | administration | 0.01328 |
|  | alleviate | 0.01244 |
|  | ameliorate | 0.01202 |
|  | protective | 0.01054 |
|  | injection | 0.01033 |
|  | ferroptosis | 0.009173 |
|  | alveolar | 0.008962 |
|  | protect | 0.008646 |
|  | alpha | 0.008541 |
|  | kg | 0.008014 |
|  | respiratory | 0.007909 |
|  | dose | 0.007804 |
| **T11** | skin | 0.03581 |
|  | hmgb1 | 0.03297 |
|  | evs | 0.02054 |
|  | ifn | 0.0196 |
|  | tlr4 | 0.01886 |
|  | gamma | 0.01791 |
|  | tlr9 | 0.0157 |
|  | exosome | 0.01391 |
|  | msc | 0.01359 |
|  | il17a | 0.01286 |
|  | toll | 0.01233 |
|  | lesion | 0.01159 |
|  | alpha | 0.01149 |
|  | tlr2 | 0.01001 |
|  | polarization | 0.009593 |
|  | differentiation | 0.009488 |
|  | vesicle | 0.008329 |
|  | nf | 0.007697 |
|  | stem | 0.007697 |
|  | il17 | 0.007592 |
| **T12** | thrombi | 0.04771 |
|  | thrombus | 0.04582 |
|  | venous | 0.03787 |
|  | fibrin | 0.0356 |
|  | stroke | 0.03547 |
|  | clot | 0.02891 |
|  | dvt | 0.0221 |
|  | vte | 0.02146 |
|  | vwf | 0.01995 |
|  | platelet | 0.01579 |
|  | ischemic | 0.01415 |
|  | deep | 0.01402 |
|  | arterial | 0.01389 |
|  | lysis | 0.009855 |
|  | plasminogen | 0.008972 |
|  | thromboembolism | 0.00872 |
|  | fibrinolysis | 0.00872 |
|  | occlusion | 0.008215 |
|  | mechanical | 0.008089 |
|  | von_willebrand | 0.00771 |
| **T13** | chromatin | 0.02192 |
|  | kill | 0.02053 |
|  | antimicrobial | 0.0203 |
|  | fungal | 0.01844 |
|  | albican | 0.01721 |
|  | defense | 0.01706 |
|  | nuclear | 0.01397 |
|  | candida | 0.01274 |
|  | granule | 0.01266 |
|  | bacteria | 0.0122 |
|  | membrane | 0.01042 |
|  | fungus | 0.008956 |
|  | require | 0.00803 |
|  | microbe | 0.007799 |
|  | microbial | 0.007722 |
|  | phagocytosis | 0.00749 |
|  | fumigatus | 0.007182 |
|  | killing | 0.007104 |
|  | essential | 0.007104 |
|  | describe | 0.006796 |
| **T14** | bacteria | 0.04046 |
|  | biofilm | 0.03707 |
|  | aureus | 0.02807 |
|  | staphylococcus_aureus | 0.01975 |
|  | aeruginosa | 0.01868 |
|  | antimicrobial | 0.01868 |
|  | coli | 0.0182 |
|  | kill | 0.01665 |
|  | killing | 0.0152 |
|  | defense | 0.01433 |
|  | bactericidal | 0.01356 |
|  | pneumoniae | 0.01269 |
|  | antibiotic | 0.0124 |
|  | resistant | 0.01201 |
|  | pneumonia | 0.01094 |
|  | resistance | 0.01065 |
|  | phagocytosis | 0.008912 |
|  | escherichia | 0.008718 |
|  | pseudomonas | 0.008234 |
|  | antibacterial | 0.008041 |
| **T15** | signature | 0.02172 |
|  | prognosis | 0.01597 |
|  | prognostic | 0.01534 |
|  | express | 0.01406 |
|  | rna | 0.0139 |
|  | predict | 0.0127 |
|  | cluster | 0.01254 |
|  | differentially | 0.01238 |
|  | network | 0.0123 |
|  | enrichment | 0.01222 |
|  | biomarker | 0.01094 |
|  | subtype | 0.01071 |
|  | proteomic | 0.01047 |
|  | database | 0.009348 |
|  | profile | 0.009109 |
|  | deg | 0.009109 |
|  | infiltration | 0.008949 |
|  | dataset | 0.008949 |
|  | set | 0.008949 |
|  | validate | 0.007831 |
| **T16** | complement | 0.02259 |
|  | drug | 0.01436 |
|  | review | 0.01422 |
|  | therapy | 0.009715 |
|  | recent | 0.008801 |
|  | discuss | 0.00866 |
|  | disorder | 0.00859 |
|  | develop | 0.008168 |
|  | current | 0.007464 |
|  | understanding | 0.007323 |
|  | approach | 0.007112 |
|  | understand | 0.007042 |
|  | highlight | 0.006268 |
|  | insight | 0.005987 |
|  | describe | 0.005846 |
|  | event | 0.005424 |
|  | future | 0.005424 |
|  | focus | 0.005354 |
|  | propose | 0.005354 |
|  | autoimmune | 0.005354 |
| **T17** | airway | 0.05053 |
|  | cf | 0.04389 |
|  | asthma | 0.03669 |
|  | chronic | 0.02784 |
|  | copd | 0.02368 |
|  | sputum | 0.02147 |
|  | neutrophilic | 0.02105 |
|  | pulmonary | 0.0187 |
|  | exposure | 0.01842 |
|  | cigarette | 0.01607 |
|  | cystic | 0.01372 |
|  | fibrosis | 0.01344 |
|  | eosinophil | 0.01164 |
|  | exacerbation | 0.01095 |
|  | s100a8 | 0.01081 |
|  | epithelial | 0.01026 |
|  | respiratory | 0.00998 |
|  | equine | 0.009564 |
|  | fluid | 0.009288 |
|  | s100a9 | 0.009288 |
| **T18** | dnase | 0.102 |
|  | nuclease | 0.04077 |
|  | degradation | 0.03564 |
|  | degrade | 0.02871 |
|  | virulence | 0.02136 |
|  | dnase1 | 0.02053 |
|  | streptococcus | 0.01956 |
|  | mutant | 0.01804 |
|  | gas | 0.01651 |
|  | serotype | 0.01041 |
|  | escape | 0.00944 |
|  | deoxyribonuclease | 0.008886 |
|  | secrete | 0.00847 |
|  | delta | 0.008331 |
|  | recombinant | 0.008193 |
|  | evasion | 0.008054 |
|  | clearance | 0.008054 |
|  | wild | 0.007915 |
|  | ability | 0.006806 |
|  | resistance | 0.006668 |
| **T19** | covid19 | 0.1389 |
|  | sar_cov2 | 0.04084 |
|  | virus | 0.03066 |
|  | respiratory | 0.02902 |
|  | syndrome | 0.02336 |
|  | viral | 0.01961 |
|  | severity | 0.01942 |
|  | influenza | 0.01721 |
|  | pneumonia | 0.01711 |
|  | coronavirus | 0.01682 |
|  | pulmonary | 0.01077 |
|  | ard | 0.009425 |
|  | critical | 0.008464 |
|  | distress | 0.008176 |
|  | alveolar | 0.00808 |
|  | mortality | 0.007695 |
|  | ill | 0.006254 |
|  | mild | 0.006062 |
|  | organ | 0.005966 |
|  | failure | 0.00587 |
| **T20** | ra | 0.08946 |
|  | alpha | 0.06191 |
|  | il8 | 0.04115 |
|  | tnf | 0.03574 |
|  | arthritis | 0.02933 |
|  | rheumatoid_arthritis | 0.02882 |
|  | il6 | 0.02291 |
|  | il1_beta | 0.02052 |
|  | synovial | 0.01976 |
|  | joint | 0.01876 |
|  | acpa | 0.01473 |
|  | fluid | 0.01423 |
|  | bone | 0.009826 |
|  | necrosis | 0.0097 |
|  | pro | 0.008693 |
|  | elisa | 0.008693 |
|  | il1 | 0.008316 |
|  | csf | 0.007938 |
|  | secretion | 0.007938 |
|  | interleukin | 0.007813 |
| **T21** | pmn | 0.1358 |
|  | parasite | 0.02955 |
|  | polymorphonuclear | 0.02955 |
|  | bovine | 0.02809 |
|  | sperm | 0.02776 |
|  | et | 0.01825 |
|  | electron | 0.008402 |
|  | expose | 0.008066 |
|  | phagocytosis | 0.008066 |
|  | leukocyte | 0.007954 |
|  | co | 0.00773 |
|  | leishmania | 0.00773 |
|  | scan | 0.007283 |
|  | exposure | 0.007171 |
|  | immunofluorescence | 0.006835 |
|  | ne | 0.006835 |
|  | confirm | 0.006724 |
|  | cattle | 0.006724 |
|  | stage | 0.006612 |
|  | kill | 0.006612 |
| **T22** | platelet | 0.1548 |
|  | coagulation | 0.04355 |
|  | thrombin | 0.03671 |
|  | generation | 0.02291 |
|  | procoagulant | 0.01729 |
|  | tf | 0.01595 |
|  | thrombotic | 0.01494 |
|  | selectin | 0.0137 |
|  | endothelial | 0.01303 |
|  | aggregation | 0.01236 |
|  | mp | 0.01236 |
|  | state | 0.01213 |
|  | intravascular | 0.01034 |
|  | aggregate | 0.00854 |
|  | thrombocytopenia | 0.007979 |
|  | event | 0.007979 |
|  | leukocyte | 0.007979 |
|  | prothrombotic | 0.007194 |
|  | heparin | 0.006969 |
|  | anticoagulant | 0.006969 |
| **T23** | liver | 0.07023 |
|  | deficiency | 0.02051 |
|  | wt | 0.01805 |
|  | deficient | 0.01752 |
|  | recruitment | 0.01514 |
|  | wild | 0.01426 |
|  | hepatic | 0.01391 |
|  | myeloid | 0.01347 |
|  | integrin | 0.01294 |
|  | beta | 0.01101 |
|  | murine | 0.01074 |
|  | iri | 0.009777 |
|  | ischemia_reperfusion | 0.009689 |
|  | express | 0.009337 |
|  | muscle | 0.009073 |
|  | ko | 0.008721 |
|  | leukocyte | 0.008281 |
|  | early | 0.008193 |
|  | infiltration | 0.008105 |
|  | alpha | 0.007137 |
| **T24** | bind | 0.03858 |
|  | protease | 0.02707 |
|  | acid | 0.01855 |
|  | mtdna | 0.01599 |
|  | peptide | 0.01567 |
|  | binding | 0.01418 |
|  | serine | 0.01066 |
|  | contain | 0.008959 |
|  | substrate | 0.008959 |
|  | surface | 0.008853 |
|  | site | 0.008746 |
|  | domain | 0.008001 |
|  | property | 0.007894 |
|  | membrane | 0.007575 |
|  | fibrinogen | 0.007361 |
|  | chain | 0.007361 |
|  | cytotoxicity | 0.006935 |
|  | cathepsin | 0.006722 |
|  | heparin | 0.006509 |
|  | molecule | 0.006296 |
| **T25** | endothelial | 0.08304 |
|  | vascular | 0.04557 |
|  | ap | 0.04075 |
|  | dysfunction | 0.02088 |
|  | ec | 0.01463 |
|  | antiphospholipid | 0.01364 |
|  | dose | 0.01293 |
|  | pancreatitis | 0.01123 |
|  | placebo | 0.01123 |
|  | syndrome | 0.01108 |
|  | trial | 0.01108 |
|  | ii | 0.01009 |
|  | therapy | 0.009807 |
|  | primary | 0.009665 |
|  | endothelium | 0.009524 |
|  | surface | 0.009098 |
|  | microvascular | 0.008672 |
|  | improve | 0.008672 |
|  | aps | 0.008246 |
|  | organ | 0.007962 |
| **T26** | plaque | 0.03639 |
|  | cardiac | 0.03158 |
|  | atherosclerosis | 0.03158 |
|  | coronary | 0.02707 |
|  | cardiovascular | 0.02512 |
|  | myocardial_infarction | 0.02376 |
|  | heart | 0.02271 |
|  | dsdna | 0.0188 |
|  | burn | 0.0185 |
|  | aortic | 0.0182 |
|  | artery | 0.017 |
|  | myocardial | 0.0161 |
|  | mi | 0.01565 |
|  | atherosclerotic | 0.01535 |
|  | stemi | 0.01445 |
|  | cholesterol | 0.01429 |
|  | lesion | 0.01234 |
|  | carotid | 0.01114 |
|  | site | 0.009635 |
|  | failure | 0.009485 |
| **T27** | sle | 0.09849 |
|  | lupus | 0.0397 |
|  | ll37 | 0.03509 |
|  | lupus_erythematosus | 0.03396 |
|  | autoimmune | 0.03284 |
|  | autoantibody | 0.0271 |
|  | ifn | 0.02348 |
|  | ln | 0.01487 |
|  | autoimmunity | 0.01262 |
|  | interferon | 0.01249 |
|  | autoantigen | 0.0115 |
|  | nephritis | 0.01012 |
|  | peptide | 0.009374 |
|  | dsdna | 0.008999 |
|  | self | 0.007751 |
|  | active | 0.007626 |
|  | antigen | 0.007502 |
|  | dendritic | 0.007377 |
|  | renal | 0.007127 |
|  | genetic | 0.006753 |
| **T28** | pma | 0.02418 |
|  | fluorescence | 0.01206 |
|  | myristate_acetate | 0.01107 |
|  | phorbol_myristate | 0.01074 |
|  | stimulation | 0.01054 |
|  | quantify | 0.01047 |
|  | stain | 0.009545 |
|  | mu | 0.009479 |
|  | concentration | 0.009015 |
|  | cytometry | 0.008618 |
|  | quantification | 0.008618 |
|  | chromatin | 0.008088 |
|  | detection | 0.007889 |
|  | immunofluorescence | 0.007359 |
|  | image | 0.007293 |
|  | culture | 0.006896 |
|  | fluorescent | 0.006763 |
|  | imaging | 0.006366 |
|  | stimulus | 0.005968 |
|  | obtain | 0.005107 |
| **T29** | sepsis | 0.1826 |
|  | septic | 0.04691 |
|  | organ | 0.03709 |
|  | shock | 0.02537 |
|  | survival | 0.02332 |
|  | trauma | 0.02112 |
|  | clp | 0.0198 |
|  | mortality | 0.01907 |
|  | dysfunction | 0.01877 |
|  | improve | 0.01628 |
|  | neonatal | 0.01101 |
|  | ligation | 0.01057 |
|  | peritoneal | 0.01042 |
|  | puncture | 0.01027 |
|  | cecal | 0.01013 |
|  | failure | 0.008954 |
|  | recombinant | 0.007342 |
|  | multiple | 0.007196 |
|  | murine | 0.006902 |
|  | early | 0.006609 |
| **T30** | anca | 0.0588 |
|  | aav | 0.05139 |
|  | vasculitis | 0.05139 |
|  | igg | 0.03707 |
|  | cytoplasmic | 0.02373 |
|  | positive | 0.02356 |
|  | fc | 0.01961 |
|  | iga | 0.01945 |
|  | pregnancy | 0.01895 |
|  | sera | 0.01681 |
|  | gamma | 0.0155 |
|  | antineutrophil | 0.01154 |
|  | fetal | 0.01138 |
|  | renal | 0.01121 |
|  | polyangiitis | 0.01105 |
|  | autoantibody | 0.009897 |
|  | maternal | 0.009567 |
|  | active | 0.009567 |
|  | glomerular | 0.008579 |
|  | complement | 0.007921 |

**Supplementary Table 4.** The top 10 article titles most relevant to the topic

| **Topic** | **Article** |
| --- | --- |
| **T1** | disulfiram accelerates diabetic foot ulcer healing by blocking net formation via suppressing the nlrp3/caspase-1/gsdmd pathway |
|  | neutrophil extracellular traps delay diabetic wound healing by inducing endothelial-to-mesenchymal transition via the hippo pathway |
|  | nlrp3 activation induced by neutrophil extracellular traps sustains inflammatory response in the diabetic wound |
|  | gsdmd drives canonical inflammasome-induced neutrophil pyroptosis and is dispensable for netosis |
|  | nlrp3 inflammasome activation and netosis positively regulate each other and exacerbate proinflammatory responses: implications of netosis inhibition for acne skin inflammation treatment |
|  | neutrophil extracellular traps induce glomerular endothelial cell dysfunction and pyroptosis in diabetic kidney disease |
|  | gnrh impairs diabetic wound healing through enhanced netosis |
|  | neutrophil extracellular traps are markers of wound healing impairment in patients with diabetic foot ulcers treated in a multidisciplinary setting |
|  | mfg-e8 accelerates wound healing in diabetes by regulating nlrp3 inflammasome-neutrophil extracellular traps axis |
|  | foxm1 network in association with trem1 suppression regulates net formation in diabetic foot ulcers |
| **T2** | the role of neutrophil extracellular traps in early microthrombosis and brain injury after subarachnoid hemorrhage in mice |
|  | neutrophils delay repair process in wallerian degeneration by releasing nets outside the parenchyma |
|  | neutrophil extracellular traps promote tpa-induced brain hemorrhage via cgas in mice with stroke |
|  | neutrophil extracellular traps exacerbate secondary injury via promoting neuroinflammation and blood-spinal cord barrier disruption in spinal cord injury |
|  | neutrophils and neutrophil extracellular traps cause vascular occlusion and delayed cerebral ischemia after subarachnoid hemorrhage in mice |
|  | inhibition of neutrophil extracellular trap formation ameliorates neuroinflammation and neuronal apoptosis via sting-dependent ire1α/ask1/jnk signaling pathway in mice with traumatic brain injury |
|  | smart liposomal nanocarrier enhanced the treatment of ischemic stroke through neutrophil extracellular traps and cyclic guanosine monophosphate-adenosine monophosphate synthase-stimulator of interferon genes (cgas-sting) pathway inhibition of ischemic penumbra |
|  | neutrophil extracellular traps released by neutrophils impair revascularization and vascular remodeling after stroke |
|  | spleen tyrosine kinase facilitates neutrophil activation and worsens long-term neurologic deficits after spinal cord injury |
|  | neutrophil extracellular traps increased by hyperglycemia exacerbate ischemic brain damage |
| **T3** | citrullinated histone h3, a biomarker for neutrophil extracellular trap formation, predicts the risk of mortality in patients with cancer |
|  | netosis in psoriatic arthritis: serum mpo-dna complex level correlates with its disease activity |
|  | immunoaging - the effect of age on serum levels of net biomarkers in men: a pilot study |
|  | predictive value of the neutrophil-to-lymphocyte ratio for treatment response in patients diagnosed with definite or probable autoimmune encephalitis/encephalopathy |
|  | increased citrullinated histone h3 levels in the early post-resuscitative period are associated with poor neurologic function in cardiac arrest survivors-a prospective observational study |
|  | neutrophil extracellular traps predict postoperative pulmonary complications in paediatric patients undergoing parental liver transplantation |
|  | increased circulating levels of neutrophil extracellular traps during cardiopulmonary bypass |
|  | predictive value of neutrophil extracellular trap components for 28-day all-cause mortality in patients with cardiac arrest: a pilot observational study |
|  | determining prognosis in canine sepsis by bedside measurement of cell-free dna and nucleosomes |
|  | neutrophil side fluorescence: a new indicator for predicting the severity of patients with bronchiectasis |
| **T4** | activation of conventional protein kinase c (pkc) is critical in the generation of human neutrophil extracellular traps |
|  | stimulation of the class-a scavenger receptor induces neutrophil extracellular traps (nets) by erk dependent nox2 and romo1 activation |
|  | oxidized ldl induced extracellular trap formation in human neutrophils via tlr-pkc-irak-mapk and nadph-oxidase activation |
|  | pharmacological inhibition of sodium-calcium exchange activates nadph oxidase and induces infection-independent netotic cell death |
|  | (+)-borneol inhibits the generation of reactive oxygen species and neutrophil extracellular traps induced by phorbol-12-myristate-13-acetate |
|  | reactive oxygen species-induced activation of erk and p38 mapk mediates pma-induced nets release from human neutrophils |
|  | formation of neutrophil extracellular traps in mitochondrial dna-deficient cells |
|  | alkaline ph promotes nadph oxidase-independent neutrophil extracellular trap formation: a matter of mitochondrial reactive oxygen species generation and citrullination and cleavage of histone |
|  | streptococcus suis serotype 2 stimulates neutrophil extracellular traps formation via activation of p38 mapk and erk1/2 |
|  | two-in-one: uv radiation simultaneously induces apoptosis and netosis |
| **T5** | arbutin alleviates intestinal colitis by regulating neutrophil extracellular traps formation and microbiota composition |
|  | neutrophil extracellular traps impair intestinal barrier function during experimental colitis |
|  | neutrophil extracellular trap density increases with increasing histopathological severity of crohn's disease |
|  | inhibition of hmgb1 improves experimental mice colitis by mediating nets and macrophage polarization |
|  | effect mechanism investigation of herb-partitioned moxibustion on relieving colon inflammation in crohn disease rats based on neutrophil extracellular traps |
|  | dclk1 isoforms and aberrant notch signaling in the regulation of human and murine colitis |
|  | polyphenol-rich diet mediates interplay between macrophage-neutrophil and gut microbiota to alleviate intestinal inflammation |
|  | dihydromyricetin ameliorates experimental ulcerative colitis by inhibiting neutrophil extracellular traps formation via the hif-1 α /vegfa signaling pathway |
|  | microbiota metabolite butyrate constrains neutrophil functions and ameliorates mucosal inflammation in inflammatory bowel disease |
|  | histones of neutrophil extracellular traps directly disrupt the permeability and integrity of the intestinal epithelial barrier |
| **T6** | the crosstalk between cancer cells and neutrophils enhances hepatocellular carcinoma metastasis via neutrophil extracellular traps-associated cathepsin g component: a potential therapeutic target |
|  | aso author reflections: induction of postoperative neutrophil extracellular trap formation after pancreatectomy may be a link between surgical inflammation and disease recurrence |
|  | the tumor-derived cytokine chi3l1 induces neutrophil extracellular traps that promote t cell exclusion in triple-negative breast cancer |
|  | breast cancer cell-neutrophil interactions enhance neutrophil survival and pro-tumorigenic activities |
|  | neutrophils extracellular traps inhibition improves pd-1 blockade immunotherapy in colorectal cancer |
|  | neutrophil extracellular trap-associated ceacam1 as a putative therapeutic target to prevent metastatic progression of colon carcinoma |
|  | mesothelin secretion by pancreatic cancer cells co-opts macrophages and promotes metastasis |
|  | increased neutrophil extracellular traps promote metastasis potential of hepatocellular carcinoma via provoking tumorous inflammatory response |
|  | liraglutide enhances the effect of checkpoint blockade in lung and liver cancers through the inhibition of neutrophil extracellular traps |
|  | neutrophil extracellular traps promote gastric cancer cell metastasis via the nat10-mediated n4-acetylcytidine modification of smyd2 |
| **T7** | pegylation of metal oxide nanoparticles modulates neutrophil extracellular trap formation |
|  | enzyme catalysis biomotor engineering of neutrophils for nanodrug delivery and cell-based thrombolytic therapy |
|  | neutrophil responses to sterile implant materials |
|  | is osseointegration inflammation-triggered? |
|  | sialic acid conjugate-modified liposomal dexamethasone palmitate targeting neutrophils for rheumatoid arthritis therapy: influence of particle size |
|  | nanoparticles size-dependently initiate self-limiting netosis-driven inflammation |
|  | immune cell response to orthopedic and craniofacial biomaterials depends on biomaterial composition |
|  | aluminum oxide nanowires as safe and effective adjuvants for next-generation vaccines |
|  | neutrophil extracellular trap-borne elastase prevents inflammatory relapse in intercritical gout |
|  | composite hydrogel modulates intrinsic immune-cascade neovascularization for ocular surface reconstruction after corneal chemical injury |
| **T8** | neutrophil hif-1α stabilization is augmented by mitochondrial ros produced via the glycerol 3-phosphate shuttle |
|  | atrazine hinders pma-induced neutrophil extracellular traps in carp via the promotion of apoptosis and inhibition of ros burst, autophagy and glycolysis |
|  | grass carp il-2 promotes neutrophil extracellular traps formation via inducing ros production and autophagy in vitro |
|  | dehp induces neutrophil extracellular traps formation and apoptosis in carp isolated from carp blood via promotion of ros burst and autophagy |
|  | zinc supplementation modulates nets release and neutrophils' degranulation |
|  | glucose induces metabolic reprogramming in neutrophils during type 2 diabetes to form constitutive extracellular traps and decreased responsiveness to lipopolysaccharides |
|  | polychlorinated biphenyl quinone metabolites cause neutrophil extracellular traps in mouse bone marrow neutrophils |
|  | switching to the cyclic pentose phosphate pathway powers the oxidative burst in activated neutrophils |
|  | effect of propiconazole on neutrophil extracellular traps formation: assessing the role of autophagy |
|  | selenium-rich yeast counteracts the inhibitory effect of nanoaluminum on the formation of porcine neutrophil extracellular traps |
| **T9** | mycobacterium tuberculosis drives expansion of low-density neutrophils equipped with regulatory activities |
|  | lymphocyte antigen 6 complex locus g6d downregulation is a novel parameter for functional impairment of neutrophils in aged mice |
|  | low-density granulocytes display immature cells with enhanced net formation in people living with hiv |
|  | host resistance and immune aging |
|  | dysregulated low-density granulocyte contributes to early spontaneous abortion |
|  | large-scale hematopoietic differentiation of human induced pluripotent stem cells provides granulocytes or macrophages for cell replacement therapies |
|  | neutrophil functional heterogeneity is a fixed phenotype and is associated with distinct gene expression profiles |
|  | low-density granulocytes display immature cells with enhanced net formation in people living with hiv |
|  | low-density neutrophils in healthy individuals display a mature primed phenotype |
|  | neutrophils in multiple sclerosis are characterized by a primed phenotype |
| **T10** | the effect and mechanism of lipoxin a4 on neutrophil function in lps-induced lung injury |
|  | re-du-ning injection ameliorates lps-induced lung injury through inhibiting neutrophil extracellular traps formation |
|  | c-x-c-chemokine-receptor-type-4 inhibitor amd3100 attenuates pulmonary inflammation and fibrosis in silicotic mice |
|  | methoxyeugenol protects against lung inflammation and suppresses neutrophil extracellular trap formation in an lps-induced acute lung injury model |
|  | neutrophil extracellular traps contribute to the pathogenesis of acid-aspiration-induced ali/ards |
|  | punicalin attenuates lps-induced acute lung injury by inhibiting inflammatory cytokine production and mapk/nf-κb signaling in mice |
|  | artemisia gmelinii extract attenuates particulate matter-induced neutrophilic inflammation in a mouse model of lung injury |
|  | protectin d1 protects against lipopolysaccharide-induced acute lung injury through inhibition of neutrophil infiltration and the formation of neutrophil extracellular traps in lung tissue |
|  | aspirin, but not tirofiban displays protective effects in endotoxin induced lung injury |
|  | delayed neutrophil apoptosis may enhance net formation in ards |
| **T11** | keratinocyte exosomes activate neutrophils and enhance skin inflammation in psoriasis |
|  | neutrophil extracellular traps activate lung fibroblast to induce polymyositis-related interstitial lung diseases via tlr9-mir-7-smad2 pathway |
|  | potential pathogenetic role of antimicrobial peptides carried by extracellular vesicles in an in vitro psoriatic model |
|  | il-17a expressed on neutrophil extracellular traps promotes mesenchymal stem cell differentiation toward bone-forming cells in ankylosing spondylitis |
|  | substance p promotes epidural fibrosis via induction of type 2 macrophages |
|  | ros-induced gingival fibroblast senescence: implications in exacerbating inflammatory responses in periodontal disease |
|  | hsa_circrna_103124 upregulation in crohn's disease promoted macrophage m1 polarization to maintain an inflammatory microenvironment via activation of the akt2 and tlr4/nf-?b pathways |
|  | neutrophil extracellular traps induce human th17 cells: effect of psoriasis-associated traf3ip2 genotype |
|  | tsg-6 is involved in fibrous structural remodeling after the injection of adipose-derived stem cells |
|  | anti-inflammatory and anti-fibrotic effects of human amniotic membrane mesenchymal stem cells and their potential in corneal repair |
| **T12** | neutrophil extracellular traps in ischemic stroke thrombi |
|  | stasis promotes erythrocyte adhesion to von willebrand factor |
|  | the mechanistic and structural role of von willebrand factor in endotoxemia-enhanced deep vein thrombosis in mice |
|  | detailed histological analysis of a thrombectomy-resistant ischemic stroke thrombus: a case report |
|  | massive platelet-rich thrombus formation in small pulmonary vessels in amniotic fluid embolism: an autopsy study |
|  | white blood cell subtypes and neutrophil extracellular traps content as biomarkers for stroke etiology in acute ischemic stroke clots retrieved by mechanical thrombectomy |
|  | citrullinated fibrinogen forms densely packed clots with decreased permeability |
|  | diverse thrombus composition in thrombectomy stroke patients with longer time to recanalization |
|  | traditional thrombus composition and related endovascular outcomes: catching up with the recent evidence |
|  | histone content, and thus dna content, is associated with differential in vitro lysis of acute ischemic stroke clots |
| **T13** | nuclear extrusion precedes discharge of genomic dna fibers during tunicamycin-induced neutrophil extracellular trap-osis (netosis)-like cell death in cultured human leukemia cells |
|  | novel cell death program leads to neutrophil extracellular traps |
|  | a myeloperoxidase-containing complex regulates neutrophil elastase release and actin dynamics during netosis |
|  | neutrophil extracellular traps in host defense |
|  | production of extracellular traps against aspergillus fumigatus in vitro and in infected lung tissue is dependent on invading neutrophils and influenced by hydrophobin roda |
|  | release of neutrophil extracellular traps in response to candida albicans yeast, as a secondary defense mechanism activated by phagocytosis |
|  | neutrophil extracellular trap in human diseases |
|  | the dermatophyte trichophyton rubrum induces neutrophil extracellular traps release by human neutrophils |
|  | a net outcome |
|  | neutrophil swarming delays the growth of clusters of pathogenic fungi |
| **T14** | resistance of hypervirulent klebsiella pneumoniae to both intracellular and extracellular killing of neutrophils |
|  | streptococcus pneumoniae forms surface-attached communities in the middle ear of experimentally infected chinchillas |
|  | haemophilus parainfluenzae strain atcc 33392 forms biofilms in vitro and during experimental otitis media infections |
|  | staphylococcus aureus biofilms release leukocidins to elicit extracellular trap formation and evade neutrophil-mediated killing |
|  | methicillin-resistant staphylococcus aureus bacterial nitric-oxide synthase affects antibiotic sensitivity and skin abscess development |
|  | survival of bacterial biofilms within neutrophil extracellular traps promotes nontypeable haemophilus influenzae persistence in the chinchilla model for otitis media |
|  | nontypeable haemophilus influenzae initiates formation of neutrophil extracellular traps |
|  | detailed mechanisms underlying neutrophil bactericidal activity against streptococcus pneumoniae |
|  | swimming motility mediates the formation of neutrophil extracellular traps induced by flagellated pseudomonas aeruginosa |
|  | antibodies mediate formation of neutrophil extracellular traps in the middle ear and facilitate secondary pneumococcal otitis media |
| **T15** | molecular subtyping and prognostic risk characterization of head and neck squamous cell carcinoma based on lysosome-related genes |
|  | distinct gene expression and immune features between different neutrophil extracellular trap-related osteosarcoma subtypes |
|  | the neutrophil extracellular traps-related gene signature predicts the prognosis of glioblastoma multiforme |
|  | a novel neutrophil extracellular trap signature predicts patient chemotherapy resistance and prognosis in lung adenocarcinoma |
|  | neutrophil extracellular trap is an important connection between hemodialysis and acute myocardial infarction |
|  | development of a prognostic neutrophil extracellular traps related lncrna signature for soft tissue sarcoma using machine learning |
|  | identifying differentially expressed genes and mirnas in kawasaki disease by bioinformatics analysis |
|  | a novel net-related gene signature for predicting dlbcl prognosis |
|  | identification of novel biomarkers and immune infiltration characteristics of ischemic stroke based on comprehensive bioinformatic analysis and machine learning |
|  | risk score constructed with neutrophil extracellular traps-related genes predicts prognosis and immune microenvironment in multiple myeloma |
| **T16** | neutrophil extracellular traps in autoimmune diseases: analysis of the knowledge map |
|  | global research trends and focus on the link between rheumatoid arthritis and neutrophil extracellular traps: a bibliometric analysis from 1985 to 2023 |
|  | fine-tuning neutrophil activation: strategies and consequences |
|  | a focus on the roles of histones in health and diseases |
|  | neutrophil extracellular traps in central nervous system (cns) diseases |
|  | does netosis contribute to the bacterial pathoadaption in cystic fibrosis? |
|  | bibliometric and visual analysis of neutrophil extracellular traps from 2004 to 2022 |
|  | therapeutic applications of nanoparticles targeting neutrophil and extracellular traps |
|  | bet on nets! or on how to translate basic science into clinical practice |
|  | the many roles of myeloperoxidase: from inflammation and immunity to biomarkers, drug metabolism and drug discovery |
| **T17** | neutrophil extracellular traps are found in bronchoalveolar lavage fluids of horses with severe asthma and correlate with asthma severity |
|  | neutrophil extracellular trap formation and extracellular dna in sputum of stable copd patients |
|  | acute air pollution exposure alters neutrophils in never-smokers and at-risk humans |
|  | endocrine-disrupting chemical exposure augments neutrophilic inflammation in severe asthma through the autophagy pathway |
|  | erythromycin suppresses neutrophil extracellular traps in smoking-related chronic pulmonary inflammation |
|  | e-cigarette use causes a unique innate immune response in the lung, involving increased neutrophilic activation and altered mucin secretion |
|  | neutrophil activation and netosis are the predominant drivers of airway inflammation in an ova/cfa/lps induced murine model |
|  | a lipid mediator hepoxilin a3 is a natural inducer of neutrophil extracellular traps in human neutrophils |
|  | myeloperoxidase inhibition decreases the expression of collagen and metallopeptidase in mare endometria under in vitro conditions |
|  | the in vitro inhibitory effect of sivelestat on elastase induced collagen and metallopeptidase expression in equine endometrium |
| **T18** | nuclease a (gbs0661), an extracellular nuclease of streptococcus agalactiae, attacks the neutrophil extracellular traps and is needed for full virulence |
|  | streptococcus pyogenes nuclease a (spna) mediated virulence does not exclusively depend on nuclease activity |
|  | deletion of ssna attenuates the pathogenicity of streptococcus suis and confers protection against serovar 2 strain challenge |
|  | acquisition of the sda1-encoding bacteriophage does not enhance virulence of the serotype m1 streptococcus pyogenes strain sf370 |
|  | streptococcus suis dnase ssna contributes to degradation of neutrophil extracellular traps (nets) and evasion of net-mediated antimicrobial activity |
|  | immunity to sda1 protects against infection by sda1+ and sda1- serotypes of group a streptococcus |
|  | m protein and hyaluronic acid capsule are essential for in vivo selection of covrs mutations characteristic of invasive serotype m1t1 group a streptococcus |
|  | neutrophil extracellular taps play an important role in clearance of streptococcus suis in vivo |
|  | competence-independent activity of pneumococcal enda mediates degradation of extracellular dna and nets and is important for virulence |
|  | extracellular deoxyribonuclease made by group a streptococcus assists pathogenesis by enhancing evasion of the innate immune response |
| **T19** | baricitinib treatment resolves lower-airway macrophage inflammation and neutrophil recruitment in sars-cov-2-infected rhesus macaques |
|  | high levels of neutrophil extracellular traps persist in the lower respiratory tract of critically ill patients with coronavirus disease 2019 |
|  | case report: fatal lung hyperinflammation in a preterm newborn with sars-cov-2 infection |
|  | differential activation of human neutrophils by sars-cov-2 variants of concern |
|  | iga2 antibodies against sars-cov-2 correlate with net formation and fatal outcome in severely diseased covid-19 patients |
|  | case report: a case of acute macular neuroretinopathy secondary to influenza a virus during long covid |
|  | neutrophil extracelluar traps in covid-19 |
|  | excessive neutrophils and neutrophil extracellular traps contribute to acute lung injury of influenza pneumonitis |
|  | neutrophil extracellular traps infiltrate the lung airway, interstitial, and vascular compartments in severe covid-19 |
|  | sars-cov-2-triggered neutrophil extracellular traps mediate covid-19 pathology |
| **T20** | s100a11 (calgizzarin) is released via netosis in rheumatoid arthritis (ra) and stimulates il-6 and tnf secretion by neutrophils |
|  | new pro-inflammatory cytokine il-40 is produced by activated neutrophils and plays a role in the early stages of seropositive rheumatoid arthritis |
|  | il-40: a new b cell-associated cytokine up-regulated in rheumatoid arthritis decreases following the rituximab therapy and correlates with disease activity, autoantibodies, and netosis |
|  | neutrophil-mediated carbamylation promotes articular damage in rheumatoid arthritis |
|  | igd enhances the release of neutrophil extracellular traps (nets) via fcdr in rheumatoid arthritis patients |
|  | specific increase in joint neutrophil extracellular traps and its relation to interleukin 6 in autoimmune arthritis |
|  | characterization of a synovial b cell-derived recombinant monoclonal antibody targeting stromal calreticulin in the rheumatoid joints |
|  | neutrophil extracellular trap-associated carbamylation and histones trigger osteoclast formation in rheumatoid arthritis |
|  | rheumatoid arthritis synovial fluid neutrophils drive inflammation through production of chemokines, reactive oxygen species, and neutrophil extracellular traps |
|  | andrographolide ameliorates rheumatoid arthritis by regulating the apoptosis-netosis balance of neutrophils |
| **T21** | leucocyte-derived extracellular trap formation significantly contributes to haemonchus contortus larval entrapment |
|  | bovine oviduct epithelial cells downregulate phagocytosis of sperm by neutrophils: prostaglandin e2 as a major physiological regulator |
|  | endothelin-1 downregulates sperm phagocytosis by neutrophils in vitro: a physiological implication in bovine oviduct immunity |
|  | ovulatory follicular fluid induces sperm phagocytosis by neutrophils, but oviductal fluid around oestrus suppresses its inflammatory effect in the buffalo oviduct in vitro |
|  | role of nets in the difference in host susceptibility to toxoplasma gondii between sheep and cattle |
|  | besnoitia besnoiti bradyzoite stages induce suicidal- and rapid vital-netosis |
|  | neutrophil extracellular traps as innate immune reaction against the emerging apicomplexan parasite besnoitia besnoiti |
|  | bottlenose dolphins (tursiops truncatus) do also cast neutrophil extracellular traps against the apicomplexan parasite neospora caninum |
|  | trypanosoma brucei brucei induces polymorphonuclear neutrophil activation and neutrophil extracellular traps release |
|  | adverse effects of single neutrophil extracellular trap-derived components on bovine sperm function |
| **T22** | inflammatory cytokines induce neutrophil extracellular traps interaction with activated platelets and endothelial cells exacerbate coagulation in moderate and severe essential hypertension |
|  | retracted: neutrophil extracellular traps enhance procoagulant activity in patients with essential hypertension (retracted article) |
|  | retracted: neutrophil extracellular traps exacerbate coagulation and endothelial damage in patients with essential hypertension and hyperhomocysteinemia (retracted article) |
|  | neutrophil extracellular traps induce a hypercoagulable state in glioma |
|  | p-selectin promotes neutrophil extracellular trap formation in mice |
|  | factor xii-mediated contact activation related to poor prognosis in disseminated intravascular coagulation |
|  | neutrophil extracellular traps promote thrombin generation through platelet-dependent and platelet-independent mechanisms |
|  | neutrophil extracellular traps induced by pro-inflammatory cytokines enhance procoagulant activity in nash patients |
|  | neutrophil extracellular traps induce aggregation of washed human platelets independently of extracellular dna and histones |
|  | platelet microparticles sustain autophagy-associated activation of neutrophils in systemic sclerosis |
| **T23** | the absence of extracellular cold-inducible rna-binding protein (ecirp) promotes pro-angiogenic microenvironmental conditions and angiogenesis in muscle tissue ischemia |
|  | absence of cold-inducible rna-binding protein (cirp) promotes angiogenesis and regeneration of ischemic tissue by inducing m2-like macrophage polarization |
|  | il-33 exacerbates liver sterile inflammation by amplifying neutrophil extracellular trap formation |
|  | impact of c57bl/6j and sv-129 mouse strain differences on ischemia-induced postnatal angiogenesis and the associated leukocyte infiltration in a murine hindlimb model of ischemia |
|  | histidine-rich glycoprotein alleviates liver ischemia/reperfusion injury in mice with nonalcoholic steatohepatitis |
|  | intrahepatic neutrophil accumulation and extracellular trap formation are associated with posthepatectomy liver failure |
|  | mcpip-1-mediated immunosuppression of neutrophils exacerbates acute bacterial peritonitis and liver injury |
|  | depletion of γδ t cells leads to reduced angiogenesis and increased infiltration of inflammatory m1-like macrophages in ischemic muscle tissue |
|  | neutrophil extracellular traps mediate acute liver failure in regulation of mir-223/neutrophil elastase signaling in mice |
|  | neutrophils undergo switch of apoptosis to netosis during murine fatty liver injury via s1p receptor 2 signaling |
| **T24** | individual impact of distinct polysialic acid chain lengths on the cytotoxicity of histone h1, h2a, h2b, h3 and h4 |
|  | leukocyte protease binding to nucleic acids promotes nuclear localization and cleavage of nucleic acid binding proteins |
|  | mast cell tryptase potentiates neutrophil extracellular trap formation |
|  | design of ultrasensitive probes for human neutrophil elastase through hybrid combinatorial substrate library profiling |
|  | extracellular histones identified in crocodile blood inhibit in-vitro hiv-1 infection |
|  | detection of beta-globin genes in the erythrocyte fraction in a patient with cervical cancer |
|  | polysialic acid interacts with lactoferrin and supports its activity to inhibit the release of neutrophil extracellular traps |
|  | thrombin and plasmin alter the proteome of neutrophil extracellular traps |
|  | towards better understanding of the heparin role in netosis: feasibility of using native mass spectrometry to monitor interactions of neutrophil elastase with heparin oligomers |
|  | polysialic acid modulates the binding of external lactoferrin in neutrophil extracellular traps |
| **T25** | increased adhesive potential of antiphospholipid syndrome neutrophils mediated by β2 integrin mac-1 |
|  | defibrotide inhibits antiphospholipid antibody-mediated neutrophil extracellular trap formation and venous thrombosis |
|  | neutrophil extracellular traps upregulate p21 and suppress cell cycle progression to impair endothelial regeneration after inflammatory lung injury |
|  | peroxisome proliferator activated receptor-γ agonist pioglitazone improves vascular and metabolic dysfunction in systemic lupus erythematosus |
|  | arap3 protects from excessive formylated peptide-induced microvascular leakage by acting on endothelial cells and neutrophils |
|  | systemic histone release disrupts plasmalemma and contributes to necrosis in acute pancreatitis |
|  | recombinant deoxyribonuclease i eye drops for ocular graft versus host disease: results of a randomized clinical trial |
|  | oral administration of lacticaseibacillus casei atcc393 promotes angiogenesis by enhancing neutrophil activity in a murine hind-limb ischemia model |
|  | targeting of cell-free dna by dnase i diminishes endothelial dysfunction and inflammation in a rat model of cardiopulmonary bypass |
|  | heparin improves alveolarization and vascular development in hyperoxia-induced bronchopulmonary dysplasia by inhibiting neutrophil extracellular traps |
| **T26** | neutrophil extracellular traps and fibrocytes in st-segment elevation myocardial infarction |
|  | the time course of markers of neutrophil extracellular traps in patients undergoing revascularisation for acute myocardial infarction or stable angina pectoris |
|  | neutrophil extracellular trap components associate with infarct size, ventricular function, and clinical outcome in stemi |
|  | reducing abdominal aortic aneurysm progression by blocking neutrophil extracellular traps depends on thrombus formation |
|  | neutrophil extracellular trap components and myocardial recovery in post-ischemic acute heart failure |
|  | prognostic value of culprit artery double-stranded dna in st-segment elevated myocardial infarction |
|  | coronary neutrophil extracellular trap burden and deoxyribonuclease activity in st-elevation acute coronary syndrome are predictors of st-segment resolution and infarct size |
|  | culprit site extracellular dna and microvascular obstruction in st-elevation myocardial infarction |
|  | neutrophil extracellular traps in myocardial tissue drive cardiac dysfunction and adverse outcomes in patients with heart failure with dilated cardiomyopathy |
|  | deoxyribonuclease is prognostic in patients undergoing transcatheter aortic valve replacement |
| **T27** | antibodies against human endogenous retrovirus k102 envelope activate neutrophils in systemic lupus erythematosus |
|  | neutrophil dysregulation is pathogenic in idiopathic inflammatory myopathies |
|  | neutrophils activate plasmacytoid dendritic cells by releasing self-dna-peptide complexes in systemic lupus erythematosus |
|  | coordination between innate immune cells, type i ifns and irf5 drives sle pathogenesis |
|  | netting neutrophils activate autoreactive b cells in lupus |
|  | cell death in the pathogenesis of systemic lupus erythematosus and lupus nephritis |
|  | type i interferon and neutrophil transcripts in lupus nephritis renal biopsies: clinical and histopathological associations |
|  | differential clearance mechanisms, neutrophil extracellular trap degradation and phagocytosis, are operative in systemic lupus erythematosus patients with distinct autoantibody specificities |
|  | ncf1-339 polymorphism is associated with altered formation of neutrophil extracellular traps, high serum interferon activity and antiphospholipid syndrome in systemic lupus erythematosus |
|  | neutrophil extracellular traps in systemic lupus erythematosus stimulate igg2 production from b lymphocytes |
| **T28** | bio-impedance measurement allows displaying the early stages of neutrophil extracellular traps |
|  | immunofluorescent detection of net components in paraffin-embedded tissue |
|  | a high-throughput real-time imaging technique to quantify netosis and distinguish mechanisms of cell death in human neutrophils |
|  | dna area and netosis analysis (dana): a high-throughput method to quantify neutrophil extracellular traps in fluorescent microscope images |
|  | netquant: automated quantification of neutrophil extracellular traps |
|  | a simple fluorescence assay for quantification of canine neutrophil extracellular trap release |
|  | measurement of net formation in vitro and in vivo by flow cytometry |
|  | stages of netosis development upon stimulation of neutrophils with activators of different types |
|  | single cell analysis of neutrophils nets by microscopic lspr imaging system |
|  | morphology of neutrophils during their activation and netosis: atomic force microscopy study |
| **T29** | neutrophil extracellular traps induce organ damage during experimental and clinical sepsis |
|  | neutrophil extracellular traps (nets) exacerbate severity of infant sepsis |
|  | protective effect of melatonin against polymicrobial sepsis is mediated by the anti-bacterial effect of neutrophils |
|  | neonatal net-inhibitory factor improves survival in the cecal ligation and puncture model of polymicrobial by inhibiting neutrophil extracellular traps |
|  | investigation of the pathological effects of histones, dna, and nucleosomes in a murine model of sepsis |
|  | plasmin and plasminogen prevent sepsis severity by reducing neutrophil extracellular traps and systemic inflammation |
|  | cirp increases icam-1+ phenotype of neutrophils exhibiting elevated inos and nets in sepsis |
|  | extracellular cirp and trem-1 axis promotes icam-1-rho-mediated netosis in sepsis |
|  | quantification of nets formation in neutrophil and its correlation with the severity of sepsis and organ dysfunction |
|  | matrix metalloproteinase-8 augments bacterial clearance in a juvenile sepsis model |
| **T30** | excessive neutrophil extracellular trap formation in anca-associated vasculitis is independent of anca |
|  | the presence of anti-neutrophil extracellular trap antibody in patients with microscopic polyangiitis |
|  | anti-neutrophil cytoplasmic antibody-associated vasculitis superimposed on post-streptococcal acute glomerulonephritis |
|  | myeloperoxidase anti-neutrophil cytoplasmic antibody affinity is associated with the formation of neutrophil extracellular traps in the kidney and vasculitis activity in myeloperoxidase anti-neutrophil cytoplasmic antibody-associated microscopic polyangiitis |
|  | neutrophil extracellular traps in neuropathy with anti-neutrophil cytoplasmic autoantibody-associated microscopic polyangiitis |
|  | neutrophil extracellular traps are present in immune-complex-mediated cutaneous small vessel vasculitis and correlate with the production of reactive oxygen species and the severity of vessel damage |
|  | formation and disordered degradation of neutrophil extracellular traps in necrotizing lesions of anti-neutrophil cytoplasmic antibody-associated vasculitis |
|  | elevated myeloperoxidase-dna complex levels in sera of patients with iga vasculitis |
|  | iga complexes induce neutrophil extracellular trap formation more potently than igg complexes |
|  | remission of granulomatosis with polyangiitis only after resection of a pulmonary nodule |

**Supplementary Table 5.** The genes between the research on NETs in the Medical Biosciences and IS

| **Gene Names** | **First Appearance Year** | **Topic** | **Total Frequency** |
| --- | --- | --- | --- |
| TLR4 | 2007 | T4 | 15 |
| NEUTROPHIL CYTOSOLIC FACTOR 2 | 2011 | T8 | 1 |
| INTERLEUKIN 11 | 2011 | T8 | 1 |
| MACROPHAGE STIMULATING FACTOR 1 | 2011 | T8 | 1 |
| TF | 2012 | T12 | 5 |
| FXII | 2012 | T12 | 2 |
| HIF1A | 2012 | T8 | 1 |
| MTOR | 2012 | T8 | 9 |
| ST8SIA2 | 2013 | T24 | 1 |
| NCAM1 | 2013 | T24 | 1 |
| PAD4 | 2013 | T2 | 30 |
| G6PD | 2013 | T4 | 2 |
| PAD2 | 2013 | T2 | 3 |
| VWF | 2014 | T12 | 32 |
| TLR2 | 2014 | T4 | 8 |
| APOE | 2014 | T22 | 1 |
| CTSC | 2014 | T24 | 1 |
| NF-KB | 2015 | T24 | 4 |
| SYK | 2015 | T2 | 3 |
| MAPKK | 2015 | T4 | 1 |
| SK3 | 2015 | T4 | 1 |
| FACTOR V | 2015 | T12 | 1 |
| FACTOR XI | 2015 | T12 | 1 |
| FACTOR XII | 2015 | T12 | 1 |
| PTPN22 | 2015 | T2 | 1 |
| PTEN | 2015 | T8 | 2 |
| ERK | 2015 | T4 | 9 |
| MEK | 2015 | T4 | 1 |
| NFKBIA | 2015 | T4 | 2 |
| C5A | 2015 | T22 | 1 |
| PMA | 2016 | T2 | 4 |
| PI3K DELTA | 2016 | T24 | 2 |
| MLKL | 2016 | T12 | 2 |
| TLR9 | 2016 | T8 | 6 |
| RAC1 | 2016 | T4 | 1 |
| MICROFLUIDIC DEVICE | 2016 | T28 | 1 |
| RIPK3 | 2016 | T12 | 2 |
| CXCL6 | 2016 | T24 | 1 |
| PKC | 2016 | T4 | 2 |
| RIPK1 | 2016 | T12 | 2 |
| GCP-2 | 2016 | T24 | 1 |
| CITH3/H3CIT | 2017 | T3 | 65 |
| C5 | 2017 | T12 | 1 |
| CASP1 | 2017 | T28 | 1 |
| NLRP3 | 2017 | T12 | 2 |
| SPHK | 2017 | T2 | 1 |
| H1 | 2017 | T24 | 2 |
| H2A | 2017 | T24 | 3 |
| H2B | 2017 | T24 | 3 |
| H3 | 2017 | T24 | 6 |
| C3 | 2017 | T12 | 1 |
| MCL1 | 2017 | T28 | 1 |
| Caspase 3 | 2017 | T8 | 6 |
| THERMAL SCRIBING | 2017 | T28 | 1 |
| H4 | 2017 | T24 | 3 |
| IGHV3-7 | 2017 | T24 | 1 |
| IGHV3-74 | 2017 | T24 | 1 |
| ATG5 | 2017 | T8 | 2 |
| PYCARD | 2017 | T28 | 1 |
| IL-18 | 2017 | T3 | 3 |
| AIM2 | 2017 | T28 | 1 |
| IL-1B | 2017 | T3 | 10 |
| Caspase 9 | 2018 | T8 | 3 |
| SIRT3 | 2018 | T12 | 1 |
| BCL-2 | 2018 | T8 | 3 |
| TRPM2 | 2018 | T8 | 1 |
| GLUT1 | 2018 | T8 | 1 |
| NOX2 | 2018 | T4 | 6 |
| GLUT-4 | 2018 | T2 | 2 |
| DNAJB1 | 2018 | T8 | 1 |
| MIR-146A | 2018 | T3 | 2 |
| BAX | 2018 | T8 | 4 |
| GPX1 | 2018 | T8 | 1 |
| NOX | 2018 | T4 | 3 |
| IL6 | 2018 | T3 | 4 |
| MICROFLUIDIC ASSAY | 2018 | T28 | 1 |
| HMGB1 | 2018 | T12 | 12 |
| IL-17A | 2018 | T12 | 2 |
| NCF1 | 2018 | T8 | 1 |
| MAPK1 | 2018 | T4 | 2 |
| BRONCHOALVEOLAR LAVAGE (BAL) | 2018 | T28 | 1 |
| MYD88 | 2018 | T22 | 1 |
| NCF2 | 2018 | T8 | 2 |
| CRP | 2018 | T3 | 1 |
| NADPH2 | 2018 | T4 | 1 |
| LC3B | 2018 | T8 | 2 |
| NFKBIE | 2018 | T8 | 1 |
| CXCL8 | 2018 | T8 | 1 |
| SOD1 | 2018 | T8 | 1 |
| CYBB | 2018 | T8 | 1 |
| MAPK14 | 2018 | T4 | 2 |
| SOD2 | 2018 | T12 | 1 |
| OPA1 | 2018 | T8 | 1 |
| LPS | 2019 | T28 | 1 |
| P38 MAPK | 2019 | T4 | 6 |
| ARG-1 | 2019 | T2 | 1 |
| MAPK3 | 2019 | T4 | 1 |
| HRTX | 2019 | T2 | 1 |
| GPR84 | 2019 | T4 | 1 |
| BAFF | 2019 | T3 | 1 |
| PRAK | 2019 | T8 | 1 |
| HIF-1 ALPHA | 2019 | T8 | 3 |
| RAF | 2019 | T4 | 1 |
| APP | 2019 | T3 | 1 |
| PLGF | 2019 | T2 | 1 |
| ER | 2019 | T4 | 1 |
| CXCL7 | 2019 | T22 | 3 |
| MYELOPEROXIDASE | 2019 | T3 | 1 |
| MPO | 2019 | T3 | 18 |
| CXCL4 | 2019 | T22 | 5 |
| STAT1 | 2019 | T2 | 1 |
| STING | 2019 | T2 | 9 |
| VEGF-A | 2019 | T2 | 2 |
| CD68 | 2019 | T3 | 1 |
| TPA | 2019 | T12 | 1 |
| DAG | 2019 | T4 | 1 |
| MIR-29 | 2019 | T3 | 1 |
| CD39 | 2019 | T12 | 1 |
| MIR-155 | 2019 | T2 | 1 |
| DNASE1 | 2019 | T2 | 3 |
| MYOG | 2019 | T2 | 1 |
| DNASE1L3 | 2019 | T24 | 1 |
| ER BETA | 2019 | T4 | 1 |
| TNF-Alpha | 2019 | T4 | 5 |
| CAD | 2019 | T24 | 1 |
| ENTPD1 | 2019 | T12 | 1 |
| ER ALPHA | 2019 | T4 | 1 |
| NOS2 | 2019 | T2 | 1 |
| CALCIUM IONOPHORES (CAL) | 2019 | T28 | 1 |
| MIP-2 | 2019 | T2 | 1 |
| CITRULLINATED HISTONE H3 | 2019 | T3 | 1 |
| LC3-II | 2020 | T8 | 1 |
| THBD-C.1418T | 2020 | T12 | 1 |
| PIK3R1 | 2020 | T8 | 2 |
| TNFR1 | 2020 | T8 | 1 |
| CCDC25 | 2020 | T8 | 1 |
| PADI4 | 2020 | T2 | 5 |
| IL8 | 2020 | T3 | 3 |
| FXI | 2020 | T12 | 1 |
| CYP1A1 | 2020 | T8 | 1 |
| FASL | 2020 | T8 | 2 |
| FAS | 2020 | T8 | 2 |
| BCL2 | 2020 | T8 | 1 |
| BECLIN1 | 2020 | T8 | 1 |
| IL36RN | 2020 | T2 | 1 |
| DYNEIN | 2020 | T8 | 1 |
| RAF1 | 2020 | T8 | 1 |
| DNASEI | 2020 | T24 | 1 |
| PROCR-H3 | 2020 | T12 | 1 |
| CYP3A138 | 2020 | T8 | 1 |
| CYP1C | 2020 | T8 | 1 |
| CYP1B1 | 2020 | T8 | 1 |
| PROCR-H1 | 2020 | T12 | 1 |
| CASPASE8 | 2020 | T8 | 1 |
| AKT | 2021 | T4 | 6 |
| MIR-1696 | 2021 | T4 | 1 |
| APE1 | 2021 | T28 | 1 |
| JNK | 2021 | T4 | 3 |
| AMPK/P38 | 2021 | T4 | 1 |
| INOS | 2021 | T8 | 2 |
| NRF2 | 2021 | T8 | 3 |
| GPX3 | 2021 | T4 | 1 |
| PCNA | 2021 | T28 | 1 |
| ELANE | 2021 | T2 | 3 |
| ELA2 | 2021 | T3 | 1 |
| S100A8 | 2021 | T3 | 5 |
| S100A9 | 2021 | T3 | 6 |
| DNA LIGASE | 2021 | T28 | 1 |
| SDF-1 | 2021 | T3 | 1 |
| CXCR4 | 2021 | T2 | 2 |
| CXCL12 | 2021 | T3 | 1 |
| CXCL10 | 2021 | T3 | 2 |
| SR-A | 2021 | T4 | 1 |
| COX-2 | 2021 | T3 | 1 |
| COL1A2 | 2021 | T24 | 1 |
| STAB2 | 2021 | T12 | 1 |
| TGF-Beta | 2021 | T4 | 2 |
| CD31 | 2021 | T24 | 1 |
| TLR1 | 2021 | T3 | 1 |
| CD177 | 2021 | T24 | 1 |
| CCL7 | 2021 | T3 | 1 |
| REDD1 | 2021 | T8 | 1 |
| PARP | 2021 | T28 | 1 |
| NFIL3 | 2021 | T8 | 1 |
| IL-6 | 2021 | T3 | 3 |
| PFKM | 2021 | T4 | 1 |
| IL10 | 2021 | T3 | 1 |
| PFKP | 2021 | T4 | 1 |
| PFLK | 2021 | T4 | 1 |
| IL12 | 2021 | T4 | 1 |
| PI3K | 2021 | T4 | 4 |
| PPBP | 2021 | T3 | 1 |
| H3F3B | 2021 | T3 | 1 |
| TAK1 | 2022 | T4 | 1 |
| PDGFBB | 2022 | T3 | 1 |
| VCAM | 2022 | T22 | 1 |
| VEGFA | 2022 | T3 | 1 |
| P110 DELTA | 2022 | T4 | 1 |
| ULK1 | 2022 | T8 | 2 |
| PF4 | 2022 | T22 | 1 |
| PYGL | 2022 | T8 | 1 |
| ORM1 | 2022 | T12 | 2 |
| VEGF | 2022 | T3 | 1 |
| TRSP | 2022 | T8 | 1 |
| ORAI2 | 2022 | T4 | 1 |
| ORAI1 | 2022 | T4 | 1 |
| PI3K BETA | 2022 | T24 | 1 |
| PKM2 | 2022 | T2 | 1 |
| TRAIL | 2022 | T3 | 1 |
| RAC GTPASE | 2022 | T24 | 1 |
| P-SELECTIN | 2022 | T22 | 1 |
| ACE2 | 2022 | T12 | 1 |
| IL22 | 2022 | T3 | 1 |
| ZNT8A | 2022 | T2 | 1 |
| HYPOXIA INDUCIBLE-FACTOR 1 | 2022 | T8 | 1 |
| HO-1 | 2022 | T8 | 1 |
| BCR-ABL1 | 2022 | T4 | 1 |
| BPI | 2022 | T3 | 1 |
| HGF | 2022 | T3 | 1 |
| C5B-9 | 2022 | T12 | 1 |
| CCL2 | 2022 | T2 | 2 |
| GMCSF | 2022 | T8 | 1 |
| GDF15 | 2022 | T3 | 1 |
| CCL20 | 2022 | T8 | 1 |
| GCSF | 2022 | T8 | 1 |
| GADA | 2022 | T2 | 1 |
| G-CSF | 2022 | T3 | 1 |
| CD11B | 2022 | T2 | 1 |
| CD200R1L | 2022 | T24 | 1 |
| DNASE-I | 2022 | T12 | 1 |
| DECTIN-1 | 2022 | T2 | 1 |
| DAD1 | 2022 | T8 | 1 |
| CD66B | 2022 | T12 | 1 |
| CX3CL1 | 2022 | T3 | 1 |
| CTAK | 2022 | T3 | 1 |
| COX2 | 2022 | T8 | 1 |
| IA2A | 2022 | T2 | 1 |
| IFNG | 2022 | T8 | 1 |
| HEMO OXYGENASE 1 | 2022 | T8 | 1 |
| IL-17 | 2022 | T12 | 1 |
| MMP-9 | 2022 | T3 | 4 |
| MIP1 ALPHA | 2022 | T3 | 1 |
| MIF | 2022 | T2 | 1 |
| MAPK | 2022 | T4 | 5 |
| IL-10 | 2022 | T4 | 1 |
| ALOX12 | 2022 | T8 | 1 |
| JAK2 | 2022 | T22 | 3 |
| IRF5 | 2022 | T28 | 1 |
| INF-GAMMA | 2022 | T12 | 1 |
| IL33 | 2022 | T3 | 1 |
| CD80 | 2022 | T4 | 1 |
| IL3 | 2022 | T8 | 1 |
| IL17A | 2022 | T2 | 2 |
| AMPK | 2022 | T8 | 1 |
| APP110 | 2022 | T3 | 1 |
| IL-22 | 2022 | T12 | 1 |
| DNA-MPO | 2023 | T8 | 1 |
| SDC1 | 2023 | T3 | 1 |
| LY6G | 2023 | T2 | 1 |
| CD21 | 2023 | T2 | 1 |
| CD3 | 2023 | T3 | 1 |
| LCN2 | 2023 | T2 | 1 |
| ADAMTS13 | 2023 | T2 | 1 |
| BASIGIN | 2023 | T8 | 1 |
| ASK1 | 2023 | T2 | 1 |
| NLRP1 | 2023 | T2 | 1 |
| CYT C | 2023 | T8 | 1 |
| LCIII | 2023 | T8 | 1 |
| ELISAS | 2023 | T28 | 1 |
| GSK-3 | 2023 | T8 | 1 |
| LC3 | 2023 | T8 | 1 |
| CCL5 | 2023 | T2 | 2 |
| RAGE | 2023 | T24 | 1 |
| MMP8 | 2023 | T2 | 1 |
| IL-8 | 2023 | T3 | 2 |
| QPCR | 2023 | T28 | 1 |
| GCN5L1 | 2023 | T8 | 1 |
| IRE1 ALPHA | 2023 | T2 | 1 |
| CD8 | 2023 | T3 | 1 |
| CD15 | 2023 | T3 | 1 |
| LC-MS/MS | 2023 | T28 | 1 |
| TLR7 | 2024 | T22 | 1 |
| VDR | 2024 | T8 | 1 |
| CCL3 | 2024 | T3 | 1 |
| TNFAIP3 | 2024 | T8 | 1 |
| NFKBIZ | 2024 | T8 | 1 |
| GPR30 | 2024 | T2 | 1 |
| MERTK | 2024 | T4 | 1 |
| JDP2 | 2024 | T8 | 1 |
| PDK2 | 2024 | T4 | 1 |
| PDK4 | 2024 | T4 | 1 |
| IL-2 | 2024 | T8 | 1 |
| ICAM1 | 2024 | T3 | 1 |
| PLCB1 | 2024 | T8 | 1 |
| PLCG2 | 2024 | T8 | 1 |
| HDACS | 2024 | T2 | 1 |
| GSDME | 2024 | T8 | 1 |
| CF-DNA | 2024 | T2 | 2 |
| FOSL2 | 2024 | T8 | 1 |
| GAP-43 | 2024 | T2 | 1 |
| RANK | 2024 | T4 | 1 |
| RANKL | 2024 | T4 | 1 |
| ERK1/2 | 2024 | T4 | 2 |
| DUSP6 | 2024 | T8 | 1 |
| DRP1 | 2024 | T8 | 1 |
| SIRT1 | 2024 | T2 | 1 |
| CYBA | 2024 | T3 | 1 |
| CX3CR1 | 2024 | T2 | 1 |
| CLEC7A | 2024 | T8 | 1 |
| PTX3 | 2024 | T3 | 1 |
| SP1 | 2025 | T2 | 1 |
| CIRP | 2025 | T2 | 1 |

# Supplementary Figures


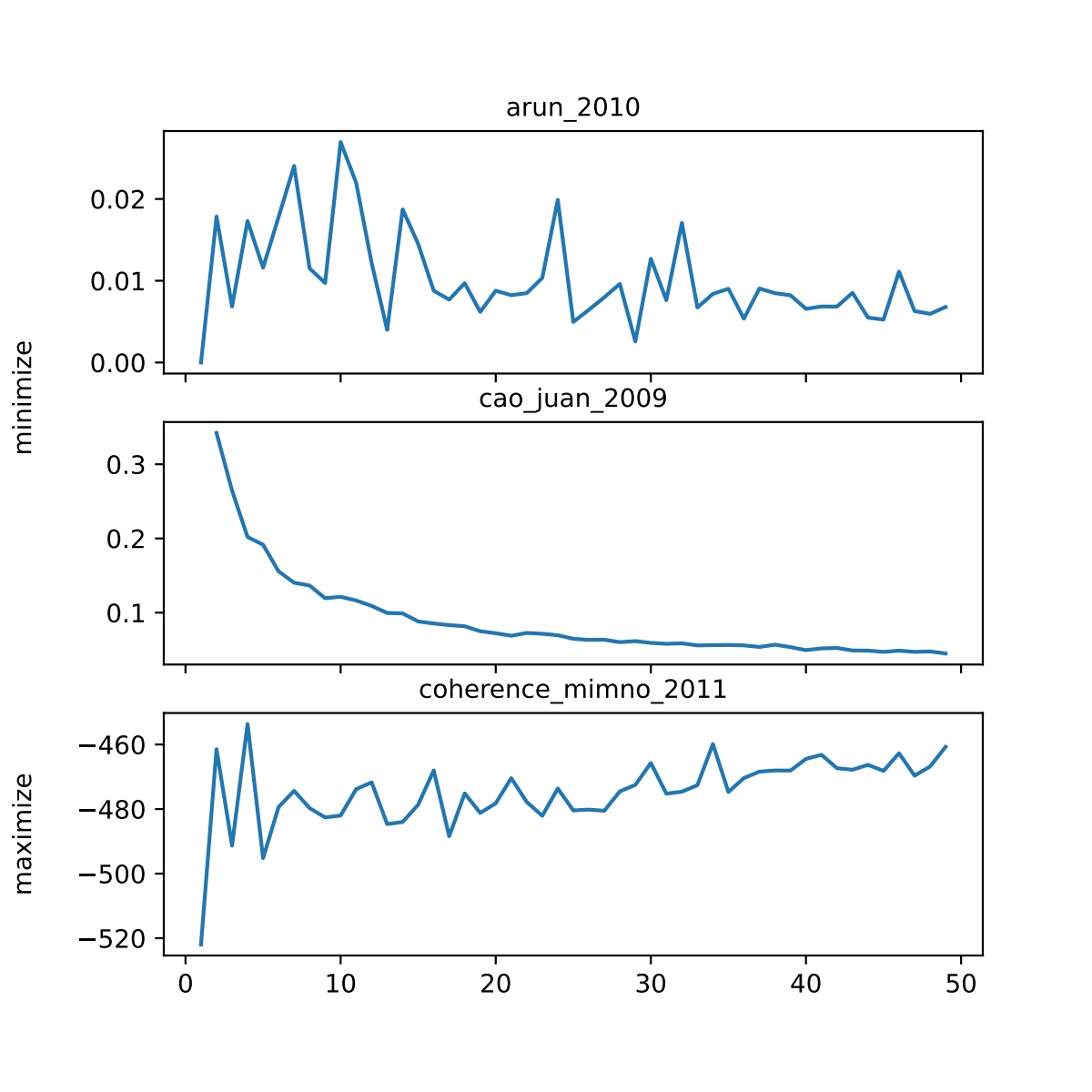


**Supplementary Figure 1.** The number of topics in LDA topic modeling is determined


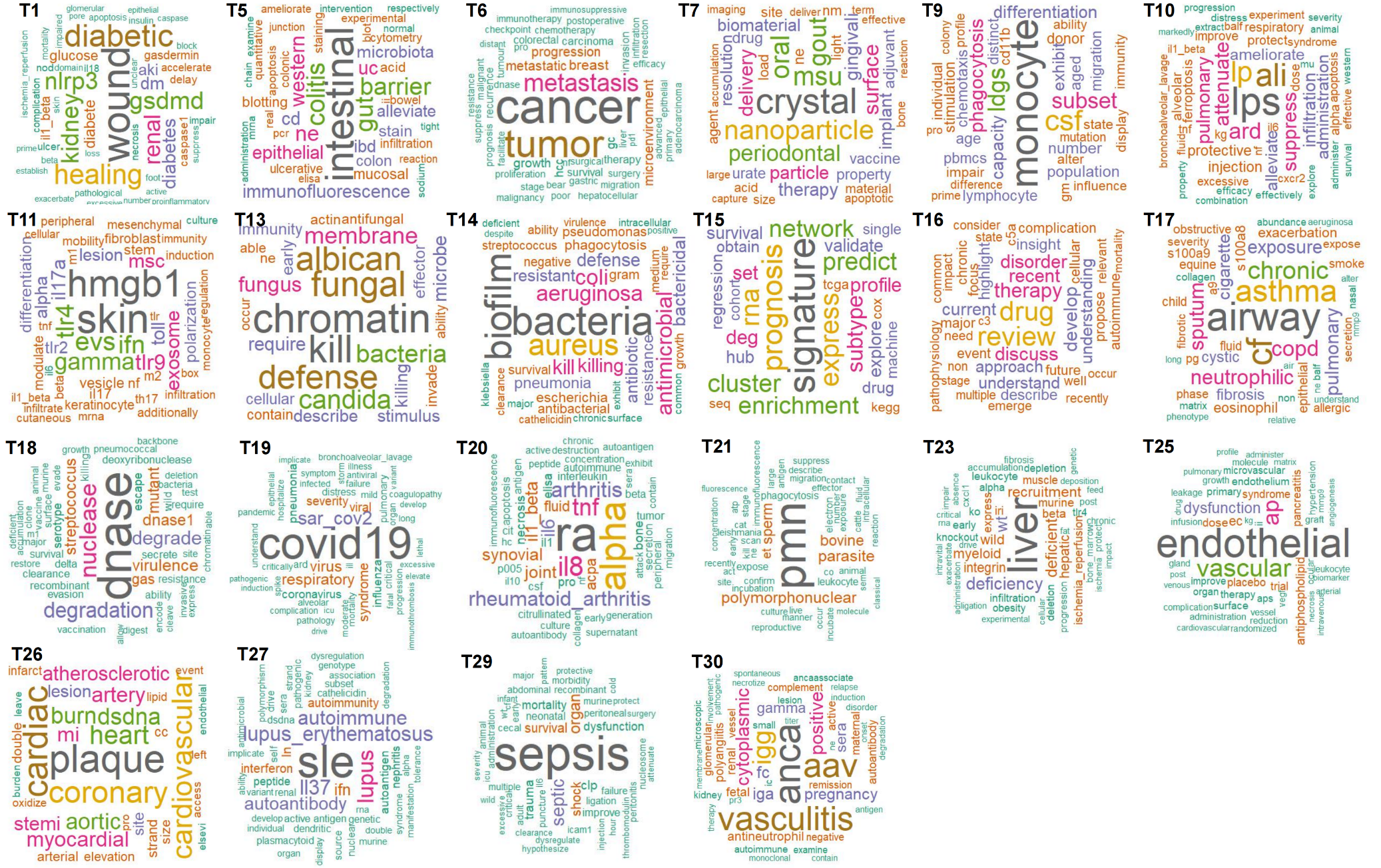


**Supplementary Figure 2.** World cloud of the role of NETs in diseases
